# Effectiveness of Digital Tools on Lifestyle & Health-Related Outcomes Across the Full Continuum of the Maternal Journey: A Systematic Review

**DOI:** 10.1007/s13679-026-00723-6

**Published:** 2026-06-10

**Authors:** Dimitra P. Sigala, Priyanka Chaudhary, Theresa H. Schroder, Zoë van der Heijden, Myriam C Afeiche, Amanda E. Staiano, Maria F. Vasiloglou

**Affiliations:** 1 Nestlé Institute of Health Science, Nestlé Research, Lausanne, Switzerland; 2 Pennington Biomedical Research Center, Baton Rouge, United States of America; 3 Division of Human Nutrition & Health, Wageningen University & Research, Wageningen, Netherlands

**Keywords:** Maternal health, Digital health, Lifestyle, Preconception, Pregnancy, Postpartum, mHealth, Systematic review

## Abstract

**Background:**

Maternal health is a global public health priority and a key determinant of generational and societal well-being. Healthy lifestyle behaviors from preconception through postpartum can be promoted by mobile applications, telehealth and chatbots, but evidence on their effectiveness remains limited despite their growing acceptance and use.

**Objectives:**

To assess the effectiveness of digital tools targeting lifestyle and health-related outcomes across the maternal journey.

**Methods:**

Following the PRISMA 2020 guidelines, a comprehensive search was conducted across PubMed, Scopus, Web of Science, and CENTRAL from January 2020 to May 2025. Eligible studies involved randomized controlled trials (RCTs) with digital components targeting nutrition, physical activity, sleep, smoking, and/or alcohol consumption among women during preconception, pregnancy, or up to six months postpartum. Risk of bias was assessed using the RoB2 tool (PROSPERO: CRD420251075108).

**Results:**

Thirty-one RCTs (*n* = 7,153 women) were included, conducted during preconception (*n* = 3), pregnancy (*n* = 20), and postpartum (*n* = 15). Across interventions, reminders (64.5%), self-monitoring (58.1%), and goal-setting (48.4%) were the most commonly used behavior change features. Digital interventions showed beneficial effects related to nutrition (e.g., improved dietary intake, micronutrient supplementation), physical activity, sleep quality, and insomnia during pregnancy. Among women with overweight/obesity who used digital tools, consistent reductions in gestational weight gain were observed. In contrast, preconception and postpartum interventions demonstrated only modest or inconsistent effects, particularly for anthropometrics and metabolic outcomes. Surprisingly, very few studies reported engagement or usage statistics.

**Conclusions:**

Digital health tools may improve lifestyle behaviors during pregnancy, but evidence for sustained preconception or postpartum benefits remains limited. The heterogeneity in study design and high risk of bias drive the need for long-term RCTs with standardized measures and engagement metrics to optimize digital strategies for maternal health promotion.

**Supplementary Information:**

The online version contains supplementary material available at 10.1007/s13679-026-00723-6.

## Introduction

Maternal health refers to a woman’s health during preconception, pregnancy, childbirth, and the years immediately following pregnancy and is the cornerstone of women’s and future generations’ welfare, and an important marker of societal advancement [1, 2]. Evidence increasingly shows that adopting healthy behaviours throughout the entire reproductive continuum (i.e., starting before conception, continued during pregnancy and the postpartum periods) is considered fundamental to achieve the optimal maternal and neonatal health outcomes and promote lifelong health trajectories [3]. A wide range of adverse maternal and offspring health conditions, including gestational diabetes mellitus (GDM), preeclampsia, suboptimal fetal development, preterm birth and small-for-gestational-age infants, are linked with modifiable lifestyle factors [4], such as balanced diet [5–7], physical activity [8], adequate sleep [9], smoking abstinence [10], and no alcohol consumption [11]. Accordingly, personalized and behaviorally informed lifestyle interventions delivered at multiple reproductive stages may offer a strategic approach to maternal health optimization, particularly when aligned with routine care pathways and personalized to women’s needs.

According to the World Health Organization (WHO), digital health technologies play a pivotal role in strengthening women’s autonomy, informing decision-making, and self-management across the maternal continuum. Specifically, the term “digital health” encompasses eHealth (which includes mHealth), as well as emerging areas, such as the use of advanced computing sciences in ‘big data’, genomics, and artificial intelligence [12]. Such technologies (i.e., mobile health (mHealth) applications, telehealth, and chatbots) provide scalable, user-centered channels for personalized health support, augment self-confidence, and promote individualised, evidence-based health education. Additionally, they enable continuous, real-time remote symptom monitoring, behavior, and medication tracking, while boosting self-efficacy, and emotional support [13].

The growing acceptance and high satisfaction rates of digital health tools form a vital opportunity to upgrade and strengthen the modern perinatal care system [14, 15]. In many middle-to-high income countries (MHICs), a substantial proportion of women of childbearing age utilises mHealth applications to monitor their health status, organize doctors’ appointments, and enhance birth preparedness [14–17].

While traditional, non-digital lifestyle interventions have indicated efficacy in advancing perinatal nutrition, physical activity, BMI, and body weights -thus suggesting the feasibility and potential of maternal health programs- they are frequently constrained by well-documented recruitment and retention challenges [4, 18–20]. The integration of digital delivery was notably advanced by foundational research before 2020, such as the LIFE-Moms trials (e.g., Smart Moms and MOMFIT), which set the significance of digitally-supported interventions for managing gestational health [21, 22]. Nevertheless, while primary studies have demonstrated positive effects of lifestyle interventions, high-quality evidence specifically supporting digital lifestyle initiatives across the maternal journey, from preconception to postpartum, remains limited and fragmented. This gap is especially important considering that current clinical recommendations underline that optimal nutrition from pre-pregnancy through toddlerhood is critical for long-term health, yet these are often overlooked in practice [7].

A consolidated synthesis is therefore essential to clarify the effectiveness of these tools, inform clinical practice, update clinical guidelines, and support the development of standardized, evidence-based digital health strategies for maternal care. Hence, this systematic review aims to assess the effectiveness and characteristics of digital tools on lifestyle (i.e., nutrition, physical activity, sleep, smoking, and alcohol consumption)and health-related outcomes (i.e., fertility, breastfeeding, anemia, insomnia, metabolic and anthropometric indicators, gestational diabetes mellitus (GDM), gestational hypertension, and urinary incontinence) across the maternal journey, from preconception to postpartum.

## Materials and Methods

### Study Design

This systematic review was designed in accordance with the Preferred Reporting Items for Systematic Reviews (PRISMA) 2020 statement and reporting checklist and has been registered in the International Prospective Register of Systematic Reviews (PROSPERO) (CRD420251075108).

### Data Sources and Search Strategy

Two independent researchers (DS and MFV) conducted a comprehensive search for scientific articles in four electronic databases: PubMed, Scopus, Web of Science, and the Cochrane Central Register of Controlled Trials (CENTRAL) assessed via Cochrane Library. Search strategies were constructed to include any appropriate subject indexing (e.g., MeSH) and free-text terms related to digital tools (e.g., “digital tools”, “mobile applications”, “ehealth”), maternal stage targeted (“preconception”, “pregnancy”, “postpartum”), lifestyle factors and health-related parameters assessed (“nutrition”, “physical activity”, “insomnia”) and dimensions of user-related parameters (“engagement”, “usability”, “attrition”) to retrieve eligible studies. Boolean operators (AND, OR) and database-specific filters were applied to refine the search. Searches were restricted to studies published between 1st of January 2020 and 28th of May 2025, in any language and location. This search timeframe was established to emphasize innovative digital health interventions that reflect the notable post-pandemic shifts in technological maturity and capabilities, clinical application, and the standardized evidence-based frameworks defined by the World Health Organization [23]. The detailed research strategy can be found in the Supplementary material.

### Inclusion and Exclusion Criteria

Studies’ eligibility followed the PICOS framework (Population, Intervention, Comparison, Outcomes and Study type): (a) Population: Studies involving women of any age, ethnicity, education level and socioeconomic status and at any stage of the maternal journey, i.e., preconception, pregnancy and/or postpartum period up to 6 months (at recruitment) after delivery; (b) Intervention: Studies involving any digital (health) tools (e.g., mobile/web/online interventions/tools/applications, chatbots) aiming at enhancing lifestyle factors and health during the maternal journey, being either fully or partially digitalized; (c) Comparators: Control groups may have included no intervention, active control, treatment as usual, waitlist control, or attention (sham) controls; (d) Outcomes: Clinical or behavioral outcomes that directly relate to at least one of the five Center of Disease Control and Prevention (CDC)-defined lifestyle parameters (nutrition, physical activity, sleep, smoking, alcohol consumption [24]), as well as evidence of effectiveness. ;e) Study Design: Randomized controlled trials (RCTs). Exclusion criteria comprised studies that did not focus on the defined maternal population, as well as interventions lacking a core digital component (e.g., in-person-only programs, digital tools that do not specifically target lifestyle or health outcomes during the defined maternal timeframe). Studies were also excluded if they targeted healthcare professionals rather than maternal users or if they addressed outcomes unrelated to the above-mentioned CDC-defined lifestyle factors. Non-randomized trials were excluded.

### Data Extraction

For each study, the following data were extracted in an excel spreadsheet independently by 2 reviewers (DS, MS, TS, MCA, AS, PC, ZH): title, author(s), year of publication, setting, geographic location, trial registration, sample size. Population; maternal stage, baseline anthropometric and demographic characteristics. Intervention; digital tool name, features and type, intervention structure, lifestyle and health domain-parameter(s) assessed, time points of assessment, duration of intervention, digital literacy, engagement tactics, usability and retention, incentives and theoretical framework used. Comparators; components of the control group intervention. Outcomes; Primary and secondary outcomes, including outcome measures (e.g., questionnaires). After completion, all extracted data were compared by DS, who reviewed and resolved discrepancies. When an outcome, such as sleep, was evaluated in a single study across both the preconception and pregnancy phases or during both the pregnancy and postpartum periods, it was considered as contributing to the assessment of that outcome in each respective phase, resulting in its inclusion in the analysis for both stages. In the discussion section, analysis was restricted to main domains (e.g., nutrition) with outcomes evaluated across two or more independent studies. All findings for subdomains (e.g., restrained eating) can be found in detail in the Supplementary Material 1.

Evidence of effectiveness was defined based on the achievement of a statistically significant difference (*p* < 0.05) between intervention and control groups, as indicated by the study authors. This evaluation was applied to all primary and secondary outcomes of each RCT extracted at every reported post-intervention time point.

### Risk of Bias Assessment

The quality and risk of bias of included studies were evaluated using the Risk of Bias Assessment 2 Tool (RoB 2 Tool) for RCTs [25] by all reviewers (DS, MS, TS, MCA, AS, PC, ZH). The domains included in RoB 2 covered bias arising from the randomization process, bias due to deviations from intended interventions, bias due to missing outcome data, bias in measurement of the outcome, and bias in selection of the reported result. Any disagreements between reviewers were resolved through discussion and consensus (DS and MFV). The “traffic-light” plot of the domain-level judgements for each individual result is presented in Supplementary Material 1.

Deviations from the registered protocol.

The original protocol also intended to comprehensively assess secondary outcomes such as retention, usability, and user satisfaction; however, this was not feasible due to the limited reporting of these outcomes in the included studies. More specifically, only a minority of studies reported on the secondary endpoints, with 19.4% (6/31) providing data on user engagement and only 6.5% (2/31) reporting measures on user satisfaction.

## Results

### Study Selection Process

**Fig. 1:**
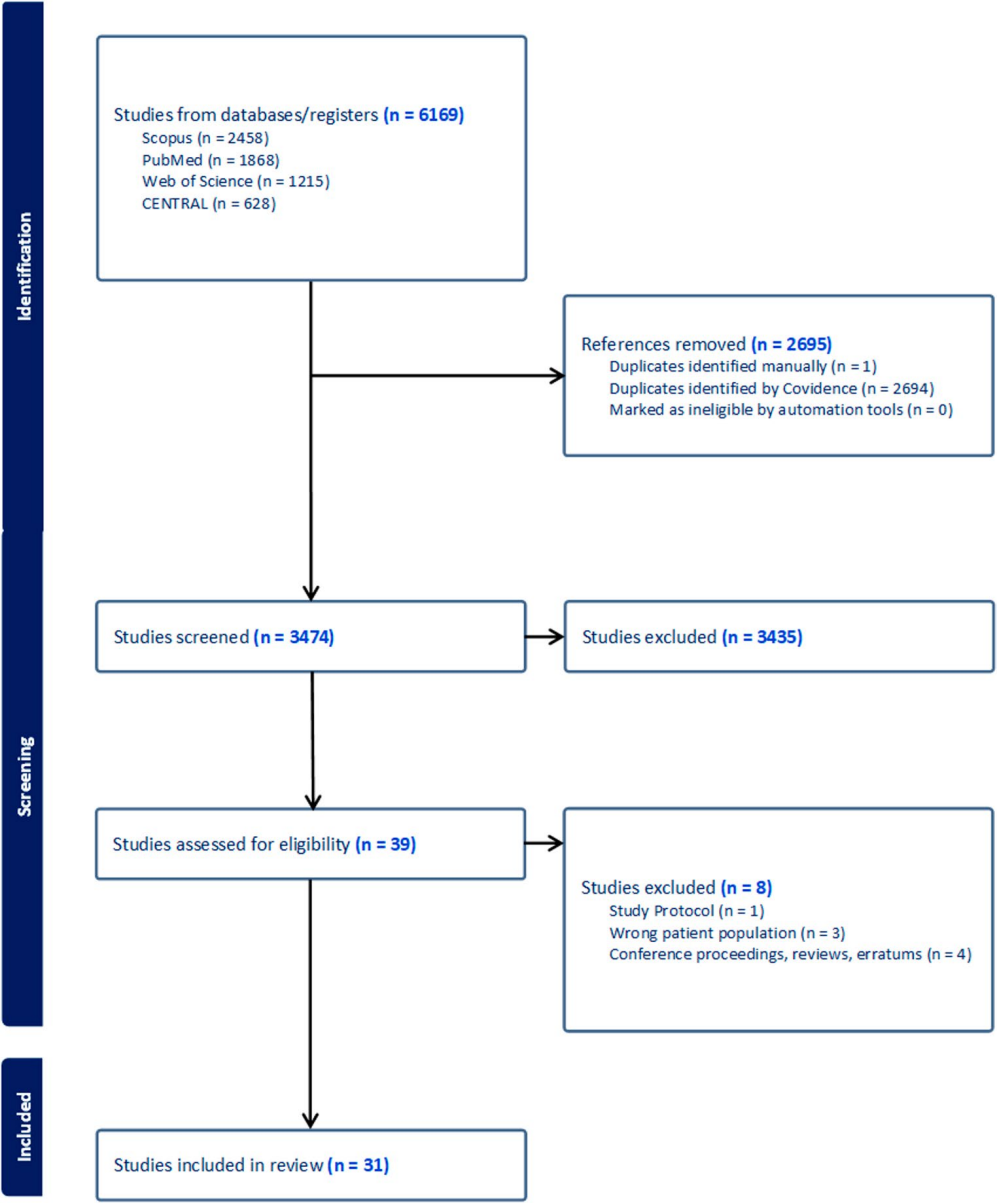
PRISMA (Preferred reporting items for systematic reviews and meta-analyses) flowchart

A comprehensive literature search was conducted in four electronic databases: Scopus, PubMed, Web of Science and CENTRAL, yielding a total of 6,169 records. Following import into COVIDENCE software, 2,695 duplicates were removed. This resulted in 3,474 unique records eligible for title and abstract screening. During screening, 3,435 studies were excluded for not meeting the inclusion criteria, leaving 39 studies underwent for full-text review. All full-text studies were retrieved, and 8 were subsequently excluded. Ultimately, 31 studies met all eligibility criteria and were included in the final qualitative synthesis (Fig. 1).

The included studies spanned across all stages of the maternal continuum, with most conducted during pregnancy (*n* = 20), followed by postpartum (*n* = 15), and a few during preconception (*n* = 3). One study included both pregnant and contemplating pregnancy populations [26], and six studies followed pregnant women into the postpartum period [27–32]. A total of 7153 women were included across the studies, with individual sample sizes ranging from 24 to 1075 participants. Interventions varied in duration, spanning from 4-weeks to 12-months. Studies were conducted across multiple countries including six in the USA [28, 33–37], five in China [29, 31, 32, 38, 39], four in the Netherlands [26, 30, 40, 41], two in Taiwan [27, 42], one in Iran [38], two in Belgium [43, 44], two in Germany [45, 46], two in Malaysia [5, 47], and one in Pakistan [48], one in Brazil [40], Indonesia [49], Spain [50], Portugal [51], Sweden [52], Singapore [53], and Australia [54]. Of the 31 studies included, 12 featured fully digitalized interventions (38.7%), while the remaining 19 were partially digitalized (61.3%) (Supplementary Material 1).

**Fig. 2:**
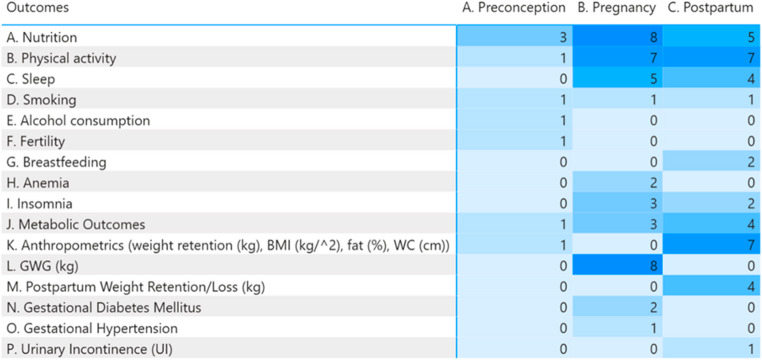
Matrix heatmap of the outcome distribution across the reproductive lifespan (Preconception. pregnancy, postpartum)

Across the CDC-defined lifestyle outcomes, nutrition and physical activity were the most prevalent intervention targets, particularly during pregnancy and postpartum. Sleep outcomes were less commonly assessed, whereas smoking and alcohol consumption were rarely measured. Anthropometric indicators including body weight, BMI, fat percentage, and waist circumference, as well as those relevant to a reproductive stage (e.g., gestational weight gain (GWG), postpartum weight retention/loss) were also frequently measured. Limited interventions on insomnia, fertility, breastfeeding, GDM, gestational hypertension, and urinary incontinence were measured. Metabolic outcomes were well represented across all maternal stages (Fig. 2).

### Preconception

Out of the 31 included studies, 3 focused specifically on the preconception period [26, 41, 47]. Two studies were conducted in the Netherlands [26, 41] and one in Malaysia [47] with sample sizes ranging from 254 to 848 participants. Mean baseline BMI varied among trials, ranging from 23.2 kg/m^2^ to 25.5 kg/m^2^, with participants classified in the overweight or obese category spanning from approximately one-quarter to nearly half. Two of the three studies included a uniform protocol comprised of a 24-week mHealth intervention targeting women undergoing IVF/ICSI, to improve nutrition [26, 41], smoking and alcohol parameters [41], compared to a lighter app version [26, 41]. Hanafiah et al. (2022) provided an e-health tool combining behavioral communication and lifestyle app-based challenges, compared to non-digital standard care [47].

### Nutrition

Improvement in dietary-related parameters was assessed in all three trials [26, 41, 47]. Participants in the Smarter Pregnancy program noted improvements in the dietary risk score (DRS) compared to the control group (β = 0.75, 95% CI 0.18–1.34) following a 24-week personalized app program, largely due to improved vegetable intake (β = 0.55, 95% CI 0.25–0.86) [26]. Likewise, participants engaging with the IVF-targeted Smarter Pregnancy platform had a notably higher decrease in DRS at the 24-week assessment point, compared to the control group (β = 0.779 for women), with effects remaining sustained till 36 weeks (β = 0.816 for women; β = 0.639 for men) [41]. In Malaysia, the Jom Mama app plus counselling (three face-to-face and three via phone sessions) did not result in dietary differences in selected food items post-intervention compared to standard care over 33-weeks, except for a significantly reduced rice portion size (71.7% vs. 50.0%, *p* = 0.001) [47].

### Physical Activity

The Jom Mama app incorporated targeted physical activity prompts (e.g., brisk walking, stair use, planks), delivered over 33 weeks. Participants reported significantly greater vigorous job-related activity than controls post intervention(IG:259.9 vs. CG: 153.8 MET/week, *p* = 0.032), with no significant effects on other types of physical activity [47].

### Smoking and Alcohol

Smoking and alcohol consumption were addressed only in the study of Oostingh et al. (2020). After 24 weeks, women in the intervention group showed a statistically significant reduction in Lifestyle Risk Score (LRS, daily smoking use and weekly alcohol consumption) compared to the control group (β = 0.108, 95% CI 0.021–0.203), whereas no significant differences were observed after 36 weeks [41].

### Health-Related Outcomes (Anthropometrics, Metabolic Outcomes, Fertility)

In Jom Mama Program, changes in weight and BMI were modest and inconsistent. After the 33-week preconception intervention, there was no significant reduction in waist circumference (WC), despite high compliance in the intervention group, i.e. all women completing six sessions and downloading the app; however, weight and BMI increases were significantly lower in the intervention group (weight: +0.8 kg, BMI: +0.3 kg/m²) compared to the control group (weight: +1.6 kg, BMI: +0.7 kg/m²). In a subgroup analysis of participants classified as obese, the intervention group gained significantly less weight than the control group (0.1 vs. 1.7 kg, *p* = 0.023) [47]. Regarding metabolic parameters, fasting blood samples, including HbA1c, TC, HDL, TGs, alongside with standardized blood pressure measurements for Systolic (SBP) and Diastolic Blood Pressure (DBP) were used in both groups, with significant findings only observed in SBP between the intervention group (107.6, SD: 14.0) and control group (104.7, SD: 10.6, *p* = 0.031) [47]. Lastly, at 52 weeks follow-up, rates of successful pregnancies did not differ significantly between intervention and control groups [41] (Supplementary Material 1).

### Pregnancy

Overall, pregnancy interventions showed consistent benefits for diet quality, physical activity and GWG among women with overweight/obesity, with mixed effects on metabolic markers. Twenty of the 31 studies focused on pregnancy -four in China [26, 29, 31, 32], three in the USA [28, 34, 37], two in the Netherlands [30, 40], two in Taiwan [27, 42], two in Iran [39, 55], and one in Brazil [48], Indonesia [49], Germany [46], Spain [50], Portugal [51], Malaysia [5], and Sweden [52], with sample sizes ranging from 24 to 306 participants and recruitment between 6th and 31st gestational weeks. The study duration varied with 5/20 trials lasting 6 weeks [5, 28, 31, 34, 37], 3/20 lasting 8 weeks [30, 51, 55], and the remaining (9/20) trials lasting 20 weeks or more, completing at or close to term. Nutrition (40%), and gestational weight gain (40%) were the most frequently reported outcomes, followed by physical activity (35%), sleep (25%), and insomnia (15%). A limited number of studies assessed metabolic outcomes (15%), anemia (10%), GDM (10%), gestational hypertension (5%), or smoking (5%). Alcohol consumption was not addressed in any study.

### Nutrition

Eight studies focused on impact on dietary intake, namely food groups (*n* = 4), macronutrient intake (*n* = 2), micronutrient intake (*n* = 4), and overall dietary management (*n* = 2). Studies analyzing changes in food group consumption showed mixed outcomes, with Vadsaria et al. (2025) observing greater intake only in the dairy food group in the app intervention group (*p* = 0.04) [48], whilst van Dijk et al. (2020) found decreased DRS (β = 0.75, 95% CI 0.18–1.34), mainly attributed to greater vegetable intake (β = 0.55, 95% CI 0.25–0.86), in the intervention group [26]. Koeryaman et al. (2023) observed higher dietary diversity (*p* = 0.005) and greater intake of vegetables and fruits (*p* < 0.001), pulses, and animal sources in the intervention compared to the control group [49].

The same authors reported also significantly greater total calorie (2502 kcal vs. 2659, *p* = 0.002), protein (70.17 g vs. 55.84 g, *p* < 0.001), and carbohydrate intake (304 g vs. 407 g, *p* = 0.02), but lower fat intake (60 g vs. 71 g, *p* < 0.001) in intervention group compared to controls (Koeryaman et al., 2023). In contrast, the dietary assessment (24 h recall) of Valença et al. (2024) found no group differences in overall energy intake [40].

Lastly, several trials focused on micronutrient intake, reporting increased iron and vitamin C intake and/or supplement use. Findings on calcium and folate intake were inconsistent. Two studies found improved iron (mg/day) and Vitamin C (mg/day) intake from the diet in healthy participants (Koeryaman et al., 2023) and those with anemia [5], after 6–12 weeks. In terms of supplements, one study reported significantly improved daily iron and vitamin D supplement intake after 24-weeks [48] but higher calcium supplement use in control group (aOR 0.59, 95% CI 0.44–0.79; *p* < 0.001) [5, 48].

### Physical Activity

Evidence on physical activity outcomes after digital interventions was mixed. Of the 7 studies reporting on physical activity, 4 focused on total physical activity; 3 of them showed statistically significant effects of the intervention in increasing participants’ total physical activity. The interventions varied in design and lasted 8 to 25 weeks. Interventions included virtual training groups and coaching as well as wearables for biofeedback and were conducted in obese and non-obese participants [50, 51, 55]. Two studies reported no significant changes on MVPA (Moderate to Vigorous Physical Activity) [50, 52] while two 8-week-studies found improvements across light (CG: 5.35 ± 3.17, IG: 12.77 ± 4.32), moderate (CG: 1.61 ± 2.58, IG: 8.31 ± 4.88), and vigorous activities (CG: 0.08 ± 0.29, IG: 0.23 ± 0.35) [55], while the other underlined better intervention outcomes in both light (*p* = 0.025), moderate (*p* = 0.005), as well as sports activities (*p* = 0.049) [51]. Sedentary behavior findings were also inconsistent: one trial (20–25 weeks) noted reduced sitting time in the intervention group contrary to controls (*p* = 0.02) [50], and another 8-week study observed increased inactivity only in the control group (*p* = 0.024) [51]. Contrarily, an 8-week PPAQ study found no differences between groups [55].

### Sleep and Insomnia

Four out of the six studies focused on improving sleep showed significant improvements in sleep parameters [28, 31, 34, 37]. Sleep duration increased by 32 min/night in the trial of Kalmbach et al. (2020) (*p* = 0.008), while Felder et al. (2020) found nonsignificant results [28]. Sleep efficiency improved in two studies [31, 34], although in one the effect was not significant after correction for multiple comparisons [31].

The effect of digital tools on insomnia in pregnancy was evaluated in three studies [28, 31, 34]. Digitally delivered CBT-I [28, 34] and digital mindfulness [31] were both effective in the reduction of insomnia severity after 18 weeks (*p* < 0.001) [34].

### Gestational Weight Gain

Overall, interventions with digital tools during pregnancy had limited [42] or non-significant effects [27, 46, 49, 52, 55] on GWG in pregnant women and one study showed significant weight loss in the intervention group [32]. However, among women with overweight/obesity, the reduction in GWG was more consistent post intervention [49, 52, 55]. In the trial of Gonzalez-Plaza et al. (2022), weekly weight gain in the intervention arm was found to be less compared to controls [50], and similarly Yang et al. (2023) showed that the intervention group experienced lower total weight gain during pregnancy [32].

### Metabolic Outcomes: Gestational Diabetes Mellitus

Evidence for improvements in metabolic markers was limited and population-specific; some studies showed significant health benefits in women with GDM, but non-significant in healthy samples. Three studies assessed metabolic parameters during pregnancy [40, 48, 52]. A 4–8-week GDM study showed better glycemic control in the intervention group (86.2% vs. 40.4%, *p* = 0.009) [40], while a 6-month trial in healthy women found no improvements in glucose or insulin resistance [52]. The incidence of GDM was evaluated in two studies and no statistically significant effects were found after digital interventions [48, 50].

### Postpartum

Overall, postpartum interventions showed consistent benefits on energy intake in populations with overweight/obesity or recent history of GDM, mixed effects on physical activity, and limited effects on anthropometric measures and metabolic outcomes. Fifteen of the 31 studies focused on postpartum; four conducted in the U.S [28, 33, 35, 36], four in China [29, 31, 32, 38], two in Belgium [43, 44], and one in the Netherlands [30], Singapore [53], Germany [45], Australia [54], and Taiwan [27]. Sample sizes ranged from 66 to 1075 participants up to 6 months postpartum. The study duration varied from 4 weeks to 12 months postpartum.

### Nutrition

Reduced energy intake was demonstrated in three studies. Bijlholt et al. (2021) observed modest decreases at 6 months postpartum (− 69 kcal/day at six months; −138 kcal/day in the obese stratum [43], and no significant change at 6 month follow-up), while interventions aimed at women with recent history of GDM showed significantly greater reductions [36, 53], ranging from approximately − 591 to − 784 kcal/day across a 6-week to 12-month period. For macronutrient intake, the study of Lim et al. (2021) revealed significant decreases in fat, carbohydrates, protein, saturated fat, and sugar consumption (*p* < 0.001) at 6 weeks and 4 months [53]. In contrast, Potzel et al. (2022) found only increases in fiber intake at six months [45], and Nicklas et al. (2024) demonstrated decreases in carbohydrate percentage and sweets intake but no changes in vegetable, fruit, whole grains, fiber, and saturated fat consumption [36].

### Physical Activity

No intervention produced significant between-group increases in MVPA [43, 45]-neither self-reported (PPAQ [33], Godin Leisure-time Exercise Questionnaire [56]), nor device-measured (accelerometer [34]), -. For sedentary time, no group differences were detected, with the exception of Nicklas et al. (2024) indicating significant decreases in the intervention group by 19.6 h/week (*p* < 0.0001) [36].

### Sleep and Insomnia

All four studies evaluating sleep outcomes assessed sleep quality. None found significant between-group differences [28, 29, 31, 33]. Contrarily, sleep duration significantly increased in women participating in digital Cognitive Behavioral Therapy treatment, who slept significantly longer than controls after childbirth by 40 min per night [28], while overall sleep quality remained unchanged. Two studies assessed insomnia during the postpartum period and found no significant between-group differences in overall ISI scores [28, 31]. Kalmbach et al. (2020) found decreased rates of sleep maintenance insomnia with CBT-I (65.9% vs. 85.4%, *p* = 0.04) following a 6-week postpartum trial [28].

### Breastfeeding

Both studies addressing breastfeeding outcomes reported solely on exclusive breastfeeding, presenting contradictory effects. Lim et al. (2021) found no significant difference in breastfeeding duration between the study arms at 4 months postpartum [53]. On the other hand, Yang et al. (2023) reported improved breastfeeding rates in the intervention compared to the control group (82.6% vs. 61.3%, *p* < 0.001) after a shorter intervention (42 days) postpartum [32].

### Metabolic Outcomes

Across 4 studies [35, 36, 45, 53] most metabolic outcomes did not show a statistically significant change. In terms of blood pressure, neither women with recent GDM [53] nor women with pre-pregnancy overweight/obesity [36] showed significant improvements post-intervention, while Herring et al. (2024) found modest but inconsistent decreases at 6 and 12 months Glycemic outcomes of the same population [35] and cardiometabolic risk indices [36] were unanimously nonsignificant.

### Anthropometric Indicators (Postpartum Weight Loss, Weight, BMI, Body Fat, Waist Circumference)

All included studies included postpartum women with excessive weight, either prior, during, or after pregnancy [27, 35, 36, 44, 54], and two also included women with normal weight [45, 53]. Intervention effects on BMI and body fat mass outcomes were modest. Two studies reported non-significant changes in fat mass [27, 44], whereas another observed a significantly higher reduction in BMI at 12 months after baseline in the intervention arm (− 1.3 kg/m², 95% CI − 1.8 to − 0.7, *p* < 0.001) [36], though between-group differences were nonsignificant (*p* = 0.13) [27, 36]. Among studies, intervention arms noted body weight change from baseline outcomes ranging from − 0.5 to − 1.6 kg at 3–6 months [35, 36, 54], with a significant within-group change in one study (− 1.6 kg, 95% CI − 3.1 to − 0.1, *p* = 0.036) [36], but absence of differences between comparison groups (*p* = 0.40). By 12 months, IG attained greater weight decreases (up to − 2.8 kg, 95% CI − 4.2 to − 1.4, *p* = 0.0002), but nonsignificance was observed between groups [36]; notably, higher engagement was linked to greater weight loss in one study (− 3.0 kg vs. + 2.4 kg, *p* = 0.01) [35]. Postpartum weight loss/retention outcomes appeared alike between IG and CG at the span of 4 to 6 months postpartum [27, 44, 53]. Weight circumference was not changed with app-interventions.

### Basic components and features of Digital Tools

All the included digital interventions were provided to participants free of charge (31/31, 100%). Most of them were delivered at an individual level (18/31, 58.1%), while only a scarce proportion involved a group-based format (3/31, 9.7%). Research-based tools were mainly used (11/31, 35.5%), whereas commercial applications and hybrid approaches (combination of commercial and research-based tools) were each described in 10/31 studies (32.3%). However, few interventions explicitly addressed accessibility needs related to low literacy, limited internet connectivity, or adaptation for resource or LMIC settings.

**Fig. 3:**
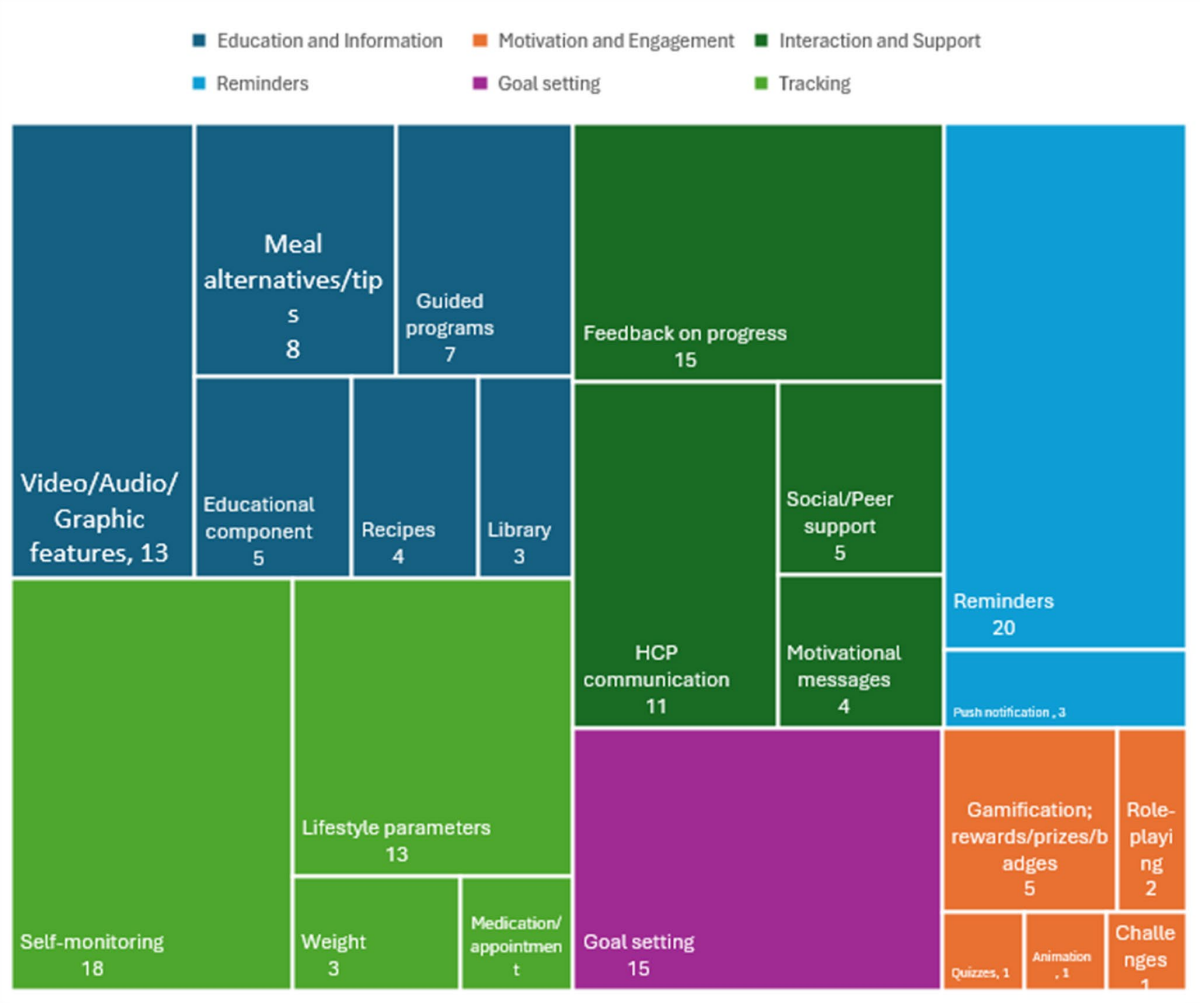
Distribution of app features in trials during the maternal journey

Among the 31 included studies, reminders (20/31, 64.5%) and self-monitoring (18/31, 58.1%) were the most prevalent features identified. Goal-setting features were also frequently found in nearly half of the studies (15/31, 48.4%). Social or peer support mechanisms (14/31, 45.2%), feedback on progress (14/31, 45.2%), as well as communication with healthcare professionals (13/31, 41.9%) were also identified. Educational and informational features were well-represented, with particular emphasis on multimedia education (13/31, 41.9%). Targeted educational features, namely structured modules (8/31, 25.8%), nutrition-related resources (meal advice (8/31, 25.8%), recipes (4/31, 12.9%)), and library information materials (4/31, 12.9%) were not as frequent. Regarding monitoring features, lifestyle parameter tracking was integrated in 13 studies (41.9%), whereas more restricted categories like weight tracking (3/31, 9.7%) and medication/appointment tracking (2/31, 6.5%) were rarely found. Motivational or engagement strategies were incorporated in a minority of studies, with gamification features (e.g. rewards, prizes, badges) observed in 4/31 studies (12.9%), role-playing games in 2/31 (6.5%), animations (2/31, 6.5%), and quizzes (1/31, 3.2%). Push notifications and motivational messages were also less common (3/31, 9.7%, each).(Fig. 3).

### Risk of Bias

The ROB 2 tool was used to evaluate the risk of bias in each study included. The traffic-light plot including all five domains of bias can be found in Supplementary Material 1. Risk of bias assessment revealed that twenty-three studies presented high risk of bias, 5 low risk of bias and three studies showed some concerns. Among studies classified as high risk of bias, the vast majority (14/23) were missing details regarding deviations from intended interventions, with subsequent deficiencies in the measurement of the outcome (13/23) and missing outcome data domains (12/23). Furthermore, four studies did not report adequately the randomization process (4/23), while 2 lacked enough information regarding the selection of the reported results (2/23). Five out of twenty-three studies identified as having a high risk of bias [45, 46, 52] also raised some concerns regarding the domains of randomization process, four from the intended intervention, one from missing outcome data, and one from selection of the reported results. Lastly, concerning studies assessed as “some concerns”, three studies suffered in the randomization process- (2/3).

## Discussion

This systematic review included thirty-one studies exploring the effectiveness of digital tools on lifestyle and health-related outcomes across the full continuum of the maternal journey (preconception, pregnancy, and postpartum), involving in total 7153 women. In the preconception phase, benefits were limited and mixed after digital interventions. When digital tools were used in pregnancy, several improvements were noted, namely macronutrient intake and vegetable consumption, dietary iron/Vitamin C intake), total physical activity, sedentary behavior, sleep quality, insomnia, with limited effects on GWG in the general population, but consistent benefits among women with overweight or obesity. Effects on energy intake, calcium and folic acid supplementation, smoking and alcohol use were inconsistent. During the postpartum period, interventions were ineffective for anthropometrics, showing only small or inconsistent improvements in energy and macronutrient intake and physical activity. On the contrary, a recent meta-analysis of RCTs found that non-digitalized lifestyle interventions targeting both dietary modification and behavior change had significantly decreased postpartum weight retention (SMD = -0.60, 95% CI -0.86 to -0.33, *p* < 0.001) [57]. In terms of the feature analysis, reminders, self-monitoring, and goal setting capabilities were the most prevalent among the trials included.

For preconception, our review aligns with Suzuki et al. (2025) on web-based interventions, showing modest benefits for physical activity and systolic blood pressure but no significant effects on other metabolic outcomes or specific dietary intake (vegetables, fruits, folic acid) during the preconception phase [56]. Similarly, Musgrave et al. (2023) reported no effectiveness of apps on energy intake, weight loss, body fat, or metabolic biomarkers compared with standard care [58]. These findings agree with recent reviews indicating that blended interventions (combining digital and conventional delivery) are more effective than digital-only approaches [59].

Regarding pregnancy, Yu et al. (2024) further emphasized that in-person dietary interventions remain the most effective strategy for managing gestational weight gain (GWG) in pregnant women with overweight or obesity [60], a conclusion supported by Wang et al. (2025), whose subgroup analysis showed GWG reductions primarily in women with overweight/obesity rather than those with GDM [61]. Collectively, these trends suggest that baseline metabolic status influences outcomes and that digital tools alone may lack sufficient intensity to sustain dietary changes during pregnancy. When hybrid models incorporating in-person counseling were applied, greater improvements in physical activity and healthy eating behaviors were observed compared to fully digital approaches [62], though effects on GWG remained limited [62, 63]. This is further highlighted by the high prevalence of partially-digitalized (61.3%) compared to fully digitalized interventions (38.7%) in our review, which underscores the reliance on blended care, thus indicating that while digital tools are notably integrated into maternal care system, human coaching and clinical visits remain a prominent component of current clinical intervention design. Moreover, our results are consistent with Han et al. (2025), who found interventions effective in enhancing physical activity [64], and partially with Sharp et al. (2022), who reported modest but consistent increases in activity measured via digital devices (steps and Moderate-to-vigorous physical activity-MVPA) [65]. In contrast, most physical activity outcomes in our review were self-reported, which may explain the heterogeneous yet generally favorable effects observed. This highlights the risk of misreporting when subjective tools such as PPAQ or IPAQ are used and underscores the need for standardized, device-based measures to improve interpretability and reduce bias in future trials.

When comparing studies on postpartum, similar to Hausvater et al. (2024) and Kwasnicki et al. (2025), who reported low to modest efficacy of digital interventions targeting diet, physical activity, and weight, our review found no notable effects on postpartum weight outcomes [66, 67]. Importantly, only one study (Herring et al., 2024) identified differential weight change by user engagement at 12 months [35] suggesting that sustained and active engagement may moderate intervention effectiveness. These findings mirror evidence from the general adult population, reinforcing that digital health interventions should complement conventional weight management strategies [68]. Overall, this points to an existent gap in postpartum digital care and the need for tailored, engagement-focused interventions, ideally delivered through hybrid models.

Beyond effectiveness our systematic review highlights moderate engagement (medium retention rates − 75%), but there is lack of clarity on how engagement is conceptualized, defined, and measured. Few studies reported engagement/usage data (6/31) and where present, most often included reminders, self-monitoring and goal-setting features [29, 50, 52]. Findings from single RCTs, such as the Healthy Moms app trial on user-engagement in relation to effectiveness in pregnancy, associated higher user-engagement with better lifestyles factor outcome (i.e. lower gestational weight gain and improved diet quality) aligning with findings from digital interventions in non-pregnant populations [69].

As mentioned in the introduction, previous systematic reviews on postpartum lifestyle interventions have not included digital tools and our review contributes to addressing this gap. However, consistent with the engagement gaps mentioned before, postpartum outcomes were modest and inconsistent, suggesting that current digital approaches might not adequately reflect and address the behavioral as well as psychological complexities of the postpartum period. However, the lack of standardized engagement metrics and theoretical framing restrains comparability and reduces understanding of the behavioral mechanisms. Recent studies indicate that blended models (i.e., digital components alongside in-person guidance) across preconception and pregnancy yield more favorable outcomes [59, 62]. Furthermore, maternal mobile health interventions appear most effective when incorporating usability-driven design, personalization, SMS communication, human interaction, and feedback [70].

### Strengths

By including only RCTs, this study assures a high level of methodological rigor, since considered as the gold standard for evaluating effectiveness. The review process followed PRISMA 2020 statement, with measures taken to mitigate bias through the quality assessment of the included trials via ROB2 tool and a robust search strategy integrating MeSH terms, were applicable. The absence of language restrictions further amplifies the inclusivity of the review, while the application of the PICOS framework and intention-to-treat analyses in most trials boost methodological transparency. Finally, this review was inclusive in respect of socioeconomic status, however majority of the trials required basic to moderate digital literacy (26/31 studies, 83.8%) (e.g., internet access, knowledge of smartphone/app use, advanced iOS operating system).

### Limitations

Firstly, the relatively broad age range (min 22.77 years, maximum 40 years), varying total sample (24 to 1075 participants), variety of digital tools and varied study durations across the maternal stages, along with lack of consistent measurements of GWG and self-reported weight data, should be taken into consideration for the reliability of outcomes. Furthermore, follow-ups were short-term (4 weeks to 9 months), and protocols were highly variable. The risk of bias constitutes also another limitation, rated mostly as high (*n* = 23/31). Finally, the absence of thorough and consistent reporting of engagement measures makes it difficult to identify adherence via user engagement data.

## Future Directions

Future research should focus on high-quality, large-scale, and long-term RCTs with further follow-up timepoints to measure sustained effects, that includes elements related to both contextual (e.g. cultural norms) and socio-demographic diversity. Whilst no geographic or individual demographic limitations were applied in the present study, only a minority (19.4%, 6/31) of the interventions involved participants Low Income and Lower Middle Income Countries (LLMICs) and also merely 3% recruited male partners [59]-in spite of the evidence of partner’s positive influence on women’s intake [71], highlighting an existing literature gap. This review identified that mHealth lifestyle interventions across the preconception period remain in infancy, constituted of a restrained number of studies, with high heterogeneity, and a significant lack of sleep outcome data, necessitating further examination to maximise their effectiveness in advancing healthy lifestyle behaviours. Also, future studies need to prioritize the inclusion of smoking and alcohol consumption data, as these variables were notably underreported in the current review. Lastly, across the entire maternal continuum, inconsistencies in definitions (e.g., GWG) and measurement methods (e.g., PPAQ versus device-measured PA) narrow comparability, thus compelling the conduct of implementation-oriented research to achieve equitable, human-centred digital maternal care. Future research should prioritize harmonized engagement definitions, validated measurement tools, and hybrid delivery strategies to optimize adherence and clinical impact [71]. Moreover, future studies should leverage co-design in the development of these interventions since the involvement of end-users can improve relevance, usability and engagement particularly across diverse cultural, socioeconomic, and geographic contexts. It is also important to acknowledge the need for interventions that can be feasibly scaled within routine maternity care. A meaningful distinction exists between primary prevention strategies targeting all women and more tailored programs designed for women with overweight or obesity; in these contexts, stand-alone digital interventions, in-person approaches, and hybrid models may each serve distinct and complementary roles. Moreover, interventions incorporating substantial digital components may support more efficient allocation of clinical resources by enabling clinicians to focus intensive support on those with greater need.

## Conclusions

This systematic review of 31 RCTs found that digital tools were partially effective in enhancing specific lifestyle and health-related parameters across the maternal stages, with the majority of the studies concerning pregnancy status. Across all stages, digital health interventions have improved nutritional outcomes, with positive sleep outcomes mostly identified during pregnancy and postpartum phases. However, effects on anthropometric outcomes appeared largely inconsistent, implying efficacy narrowed for preconception and overweight/obese-specific gestational weight gain management, with a lack of substantial impact on the postpartum population. Given that most included studies showed a high risk of bias, these findings should be interpreted with due caution. Future research should emphasize high-quality, long-term RCTs, ensuring more indicative representation of LLMIC, as well as the application of consistent measurement definitions. Studies on exploring their ecological validity and clinical application to the real world are also needed. Strengthening methodological quality and equity in this field is vital to advance the use of digital technologies across the maternal health environment to improve lifestyle and health-related outcomes. The current work highlights a gap in postpartum digital care and emphasizes the need for more personalized, engagement-driven strategies, ideally complemented by hybrid delivery approaches.

## Key References

- Koletzko B, Godfrey KM, Poston L, Szajewska H, van Goudoever JB, de Waard M, et al. Nutrition During Pregnancy, Lactation and Early Childhood and its Implications for Maternal and Long-Term Child Health: The Early Nutrition Project Recommendations. Ann Nutr Metab. 2019;74 [2]:93–106.
  - ○ Current recommendations for pregnant women, especially those living with obesity, and young children often overlook the long-term health effects of early nutrition. In this paper, updated guidance for optimal nutrition from pre-pregnancy through toddlerhood, with emphasis on long-term health outcomes is presented.
- WHO. Digital tools can help improve women’s health and promote gender equality, WHO report shows [Internet]. 2024. Available from: https://www.who.int/europe/news/item/08-03-2024-digital-tools-can-help-improve-women-s-health-and-promote-gender-equality--who-report-shows.
  - ○ Digital health technologies positively impact women’s empowerment, facilitate the achievement of gender equality, and improve health outcomes for women.
- “Wang, J., Tang, N., Jin, C., Yang, J., Zheng, X., Jiang, Q., Li, S., Xiao, N., & Zhou, X. (2025). Association of Digital Health Interventions With Maternal and Neonatal Outcomes: Systematic Review and Meta-Analysis. Journal of medical Internet research, 27, e66580. 10.2196/66580.
  - ○ Digital health interventions significantly improved excessive gestational weight gain, reduced miscarriage and preterm birth incidence, and enhanced neonatal outcomes.

## Supplementary Information

Below is the link to the electronic supplementary material.

Supplementary Material 1

## Data Availability

This study was based on previously published data and is therefore available in the original studies. All data generated in this study are included in this article and in the Appendix.
