# Supplementary material for: Effectiveness of Digital Tools on Lifestyle & Health-Related Outcomes Across the Full Continuum of the Maternal Journey: A Systematic Review
Source: Curr Obes Rep. 2026 Jun 10;15(1):45. doi: 10.1007/s13679-026-00723-6 (PMC13249648; doi:10.1007/s13679-026-00723-6)
Supplement: Supplementary file 1 — Supplementary Material 1 [file 13679_2026_723_MOESM1_ESM.docx]

**Supplementary Material 1**

**Table of Contents**

[Supplementary Table 1. PICOS criteria 3](#_Toc229153606)

[Supplementary Table 2. Search strategy 4](#_Toc229153607)

[Supplementary Table 3. Reasons for Exclusion 7](#_Toc229153608)

[Supplementary Figure 1. Matrix Heatmap of the Outcome Distribution Across the Reproductive Lifespan (Preconception. Pregnancy, Postpartum) 9](#_Toc229153609)

[Supplementary Figure 2. Traffic-Light Plot of Included Studies (n = 31) 10](#_Toc229153610)

[Supplementary Table 4. Preconception 11](#_Toc229153611)

[Supplementary Table 5. Pregnancy 14](#_Toc229153612)

[Supplementary Table 6. Postpartum 22](#_Toc229153613)

[Supplementary Table 7. Measures, Methods and Digitalization Category (Fully/Partially) of Included Lifestyle Interventions 38](#_Toc229153614)

[Supplementary Table 8. PRISMA 2020 checklist 49](#_Toc229153615)

[Supplementary Table 1. PICOS criteria 49](#_Toc229153616)

# Supplementary Table 1. PICOS criteria

|  | Inclusion Criteria | Exclusion Criteria |
| --- | --- | --- |
| P | Studies involving women of any age, ethnicity, education level and socioeconomic status and at any stage of the maternal journey, i.e., preconception, pregnancy and/or postpartum period up to 6 months (at recruitment) | Studies not focused on the defined maternal population, ; . |
| I | Studies involving digital (health) tools (e.g., mobile/web/online interventions/tools/applications, chatbots) aiming at enhancing lifestyle factors and health during the maternal journey | Studies with non-digital interventions (e.g. in-person-only programs, digital tools that do not specifically target lifestyle or health outcomes during the defined maternal timeframe, or professional targeted interventions) |
| C | Control groups may have included no intervention, active control, treatment as usual, waitlist control, or attention (sham) controls | N/A |
| O | Studies with clinical or behavioral outcomes that directly relate to at least one of the five Center of Disease Control and Prevention (CDC)-defined lifestyle parameters: nutrition, physical activity, sleep, smoking, alcohol consumption, as well as evidence of effectiveness. | Studies with outcomes unrelated to CDC lifestyle factors or focused solely on fertility, lactation, symptom tracking, gestational weight gain, or postpartum weight loss (unless linked to lifestyle factors) |
| S | Randomized controlled trials (RCTs) | Non-randomized studies |

# Supplementary Table 2. Search strategy

| Database | Searches |
| --- | --- |
| PubMed | ("Mobile Applications"[Mesh] OR "Internet-Based Intervention"[Mesh] OR "Smartphone"[Mesh] OR "digital tool*"[tiab] OR "digital health tool*"[tiab] OR "digital intervention*"[tiab] OR "web application*"[tiab] OR "mobile-based intervention*" OR "mobile based intervention*" OR "health technolog*"[tiab] OR "health app*"[tiab] OR "mobile app*"[tiab] OR "electronic tool*"[tiab] OR "online intervention*"[tiab] OR "e-health"[tiab] OR ehealth[tiab] OR "electronic health"[tiab] OR "m-health"[tiab] OR mhealth[tiab] OR "mobile health"[tiab] OR "smartphone*"[tiab] OR web[tiab] OR online[tiab] OR app[tiab] OR apps[tiab] OR application*[tiab] OR chatbot*[tiab])  AND  ("Pregnancy"[Mesh] OR "Mothers"[Mesh] OR "Prenatal Care"[Mesh] OR "Pregnancy Trimesters"[Mesh] OR "Postpartum Period"[Mesh] OR preconception[tiab]  OR "pre-conception"[tiab]   OR pregnan*[tiab] OR prenatal[tiab]  OR antenatal[tiab]  OR perinatal[tiab] OR maternal[tiab] OR "maternal journey"[tiab:~0] OR postpartum[tiab]  OR postnatal[tiab]  OR motherhood[tiab]  OR "new mother*"[tiab]  OR "expecting mother*"[tiab])  AND  ("Fertility"[Mesh] OR "Lactation"[Mesh] OR "Breast Feeding"[Mesh] OR "Nutritional Status"[Mesh] OR "Health Status"[Mesh] OR "Sleep"[Mesh] OR "Weight Gain"[Mesh] OR "Weight Loss"[Mesh] OR "Smoking"[Mesh] OR "Alcohol Drinking"[Mesh] OR fertility[tiab] OR "symptom tracking"[tiab] OR "symptom management"[tiab] OR "symptom monitor*"[tiab] OR "health tracking"[tiab] OR "monitoring symptoms"[tiab] OR lactation[tiab] OR breastfeeding[tiab] OR "breast feeding"[tiab] OR nutrition[tiab] OR diet[tiab] OR "eating habits"[tiab] OR "nutritional intake"[tiab] OR "nutritional status"[tiab] OR sleep[tiab] OR "sleep quality"[tiab] OR insomnia[tiab]  OR exercise[tiab] OR "physical activity"[tiab] OR fitness[tiab] OR "smok*"[tiab] OR "tobacco use"[tiab] OR alcohol[tiab])  AND  ("engagement"[tiab] OR "user engagement"[tiab] OR "retention"[tiab] OR "dropout"[tiab] OR "attrition"[tiab] OR "usability"[tiab] OR "user experience"[tiab] OR "user satisfaction"[tiab] OR "acceptability"[tiab] OR "feasibility"[tiab] OR "effectiveness"[tiab] OR "efficacy"[tiab])  NOT  (animals) |
| Scopus | ( TITLE-ABS-KEY ( "digital tool" OR "digital health tool" OR "digital intervention" OR "web application" OR "mobile-based intervention*" OR "mobile based intervention*" OR "health technolog*" OR "health app" OR "mobile app" OR "electronic tool" OR "online intervention" OR ehealth OR "electronic health" OR mhealth OR "mobile health" OR smartphone OR web OR online OR app OR application OR chatbot ) AND TITLE-ABS-KEY ( preconception OR pregnan* OR prenatal OR antenatal OR perinatal OR maternal OR "maternal journey" OR postpartum OR postnatal OR motherhood OR "new mother" OR "expecting mother" ) AND TITLE-ABS-KEY ( fertility OR "symptom tracking" OR "symptom management" OR "symptom monitor" OR "health tracking" OR "monitoring symptoms" OR lactation OR breastfeeding OR "breast feeding" OR nutrition OR diet OR "eating habits" OR "nutritional intake" OR "nutritional status" OR sleep OR "sleep quality" OR insomnia OR exercise OR "physical activity" OR fitness OR "smok*" OR "tobacco use" OR alcohol ) AND TITLE-ABS-KEY ( engagement OR "user engagement" OR retention OR dropout OR attrition OR usability OR "user experience" OR "user satisfaction" OR acceptability OR feasibility OR effectiveness OR efficacy ) AND NOT TITLE-ABS-KEY ( animals ) ) |
| Web of Science | TS=(("digital tool" OR "digital health tool" OR "digital intervention" OR "web application" OR "mobile-based intervention" OR "mobile based intervention" OR "health technology" OR "health app" OR "mobile app" OR "electronic tool" OR "online intervention" OR e-health OR "electronic health" OR m-health OR "mobile health" OR smartphone OR web OR online OR "app" OR "apps" OR application OR chatbot)) AND TS=(("pre-conception" OR "preconception" OR "pregnant" OR "prenatal" OR "antenatal" OR "perinatal" OR "maternal" OR "maternal journey" OR "postpartum" OR "postnatal" OR "motherhood" OR "new mother" OR "expecting mother")) AND TS=(("fertility" OR "symptom tracking" OR "symptom management" OR "symptom monitor" OR "health tracking" OR "monitoring symptoms" OR "lactation" OR "breastfeed" OR "nutrition" OR "diet" OR "eating habits" OR "nutritional intake" OR "nutritional status" OR "sleep" OR "sleep quality" OR "insomnia" OR "exercise" OR "physical activity" OR "fitness" OR "smoke" OR "tobacco use" OR alcohol)) AND TS=(("engagement" OR "user engagement" OR "retention" OR "dropout" OR "attrition" OR "usability" OR "user experience" OR "user satisfaction" OR "acceptability" OR "feasibility" OR "effectiveness" OR "efficacy")) NOT TS=("animals") |
| CENTRAL | **Identifying Digital Tools:**  **MeSH Descriptors:**  #1: Mobile Applications - 2352 hits  #2: Internet-Based Intervention - 896 hits  #3: Smartphone - 1252 hits  **Keyword Search:**  #4: Various digital tools and interventions (e.g., digital health tools, mobile apps, e-health) - 149,565 hits  **Combined Search:**  #5: Combination of #1, #2, #3, and #4 - 149,692 hits  **Maternal Health Aspects:**  **MeSH Descriptors:**  #6: Pregnancy - 32,858 hits  #7: Mothers - 3,377 hits  #8: Prenatal Care - 2,309 hits  #9: Pregnancy Trimesters - 79 hits  #10: Postpartum Period - 2,046 hits  **Keyword Search:**  #11: Terms related to maternal health (e.g., preconception, postpartum, motherhood) - 110,830 hits  **Combined Search:**  #12: Combination of #6, #7, #8, #9, #10, and #11 - 111,867 hits  **Integrating Digital Tools with Maternal Health:**  **Combined Search:**  #13: #5 AND #12 - 8,542 hits  **Additional Health Factors:**  **MeSH Descriptors:**  #14: Breast Feeding - 2,869 hits  #15: Nutritional Status - 3,681 hits  #16: Health Status - 4,935 hits  #17: Sleep - 6,196 hits  #18: Weight Gain - 3,470 hits  #19: Weight Loss - 8,741 hits  #20: Tobacco Use - 140 hits  #21: Alcohol Drinking - 5,560 hits  **Keyword Search:**  #22: Terms related to health behaviors (e.g., fertility, nutrition, exercise) - 386,385 hits  **Combined Search:**  #23: #14 OR #15 OR #16 OR #17 OR #18 OR #19 OR #22 - 393,674 hits  **Final Integration of All Aspects:**  **Combined Search:**  #24: #5 AND #12 AND #23 - 2,950 hits  **User Engagement Factors:**  **Keyword Search:**  #25: Terms related to user engagement and experience - 694,628 hits  **Combined Search:**  #26: #5 AND #12 AND #23 AND #25 - 1,569 hits  **Refining to Specific Journals:**  **Journal Filter:**  #27: journal:pt - 1,654,449 hits  **Final Combined Search:**  #28: #26 AND #27 - 721 hits  **Exclusion of Animal Studies:**  #29: #28 NOT (animal) - 711 hits |

# Supplementary Table 3. Reasons for Exclusion

| **Study** | **Reason** |
| --- | --- |
| Huang, Q., Zhong, Q., Zeng, Y., Li, Y., Wiley, J., Wang, M. P., Chen, J. L., & Guo, J. (2025). mHealth-Based Diabetes Prevention Program for Chinese Mothers With Abdominal Obesity: Randomized Controlled Trial. *JMIR mHealth and uHealth*, *13*, e47837. <https://doi.org/10.2196/47837> | Wrong patient population |
| Deruelle, P., Lelorain, S., Deghilage, S., Couturier, E., Guilbert, E., Berveiller, P., Sénat, M. V., Vayssière, C., Sentilhes, L., Perrotin, F., Gallot, D., Chauleur, C., Sananes, N., Roth, E., Luton, D., Caputo, M., Lorio, E., Chatelet, C., Couster, J., Timbely, O., … Pigeyre, M. (2020). Rationale and design of ePPOP-ID: a multicenter randomized controlled trial using an electronic-personalized program for obesity in pregnancy to improve delivery. *BMC pregnancy and childbirth*, *20*(1), 602. <https://doi.org/10.1186/s12884-020-03288-x> | Study Protocol |
| Khunti, K., Sukumar, N., Waheed, G., Gillies, C., Dallosso, H., Brough, C., Davies, M. J., Fitzpatrick, C., Gray, L. J., Highton, P., Rowlands, A., Schreder, S., Yates, T., & Saravanan, P. (2023). Structured group education programme and accompanying mHealth intervention to promote physical activity in women with a history of gestational diabetes: A randomised controlled trial. *Diabetic medicine : a journal of the British Diabetic Association*, *40*(7), e15118. <https://doi.org/10.1111/dme.15118> | Wrong patient population |
| Beleigoli, A., Andrade, A. Q., Diniz, M. F., & Ribeiro, A. L. (2020). Personalized Web-Based Weight Loss Behavior Change Program With and Without Dietitian Online Coaching for Adults With Overweight and Obesity: Randomized Controlled Trial. *Journal of medical Internet research*, *22*(11), e17494. <https://doi.org/10.2196/17494> | Wrong patient population |
| Rawal, Shristi & White, Jenna & Nepal, Jyoti & Subedi, Shanti & Daneault, Jean-Francois & Shakya, Prabin & Shrestha, Abha & Shrestha, Archana. (2023). PTFS01-05-23 Efficacy of a Mobile App Intervention for Management of Gestational Diabetes: An Exploratory Randomized Controlled Trial. Current Developments in Nutrition. 7. 101127. 10.1016/j.cdnut.2023.101127. | Conference proceedings, reviews, erratums |
| D A Kalmbach, A Cuamatzi-Castelan, C V Tonnu, T Roth, R Sangha, L M Swanson, L M O’Brien, C L Drake, 0470 A Randomized Controlled Trial of Digital Cognitive Behavioral Therapy for Insomnia in Pregnant Women, *Sleep*, Volume 43, Issue Supplement_1, April 2020, Page A180, <https://doi.org/10.1093/sleep/zsaa056.467> | Conference proceedings, reviews, erratums |
| Saxon CE, Seely EW, Bertin KB, et al. Self-Efficacy and Readiness to Change Among Women with Recent Gestational Diabetes Engaging in a Web-Based Lifestyle Intervention: The Balance After Baby Intervention Trial. *American Journal of Lifestyle Medicine*. 2023;0(0). doi:[10.1177/15598276231155147](https://doi.org/10.1177/15598276231155147), | Conference proceedings, reviews, erratums; Dimitra Sigala (2025-06-19 21:33:52)(Select): Abstracts from the 2020 Annual Meeting of the Society of General Internal Medicine  *Abstracts from the 2021 Annual Meeting of the Society of General Internal Medicine. (2021). Journal of General Internal Medicine, 36(1), 1-469.* [*https://doi.org/10.1007/s11606-021-06830-5*](https://doi.org/10.1007/s11606-021-06830-5) |
| Feasibility of an mhealth postpartum lifestyle intervention for women with cardiometabolic risk pre-and mid-covid: the fit after baby pilot randomized controlled trial. Nicklas, JM; Leiferman, J; Pyle, L; Soares, A; Bull, S; Tong, S; Barbour, LA 2021; 36( SUPPL 1): S178 2021 | Conference proceedings, reviews, erratums; Dimitra Sigala (2025-06-19 21:35:11)(Select): Abstracts from the 2020 Annual Meeting of the Society of General Internal Medicine  *("Abstracts from the 2021 Annual Meeting of the Society of General Internal Medicine," 2021)* |

# Supplementary Figure 1. Matrix Heatmap of the Outcome Distribution Across the Reproductive Lifespan (Preconception. Pregnancy, Postpartum)


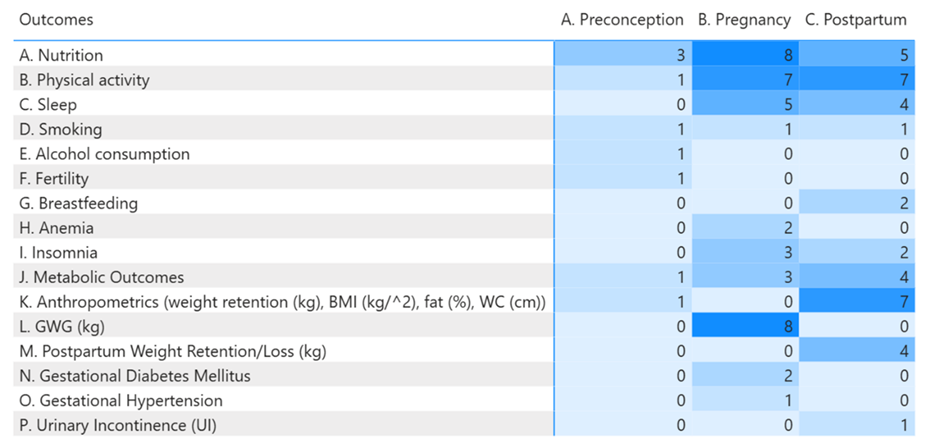


# Supplementary Figure 2. Traffic-Light Plot of Included Studies (n = 31)


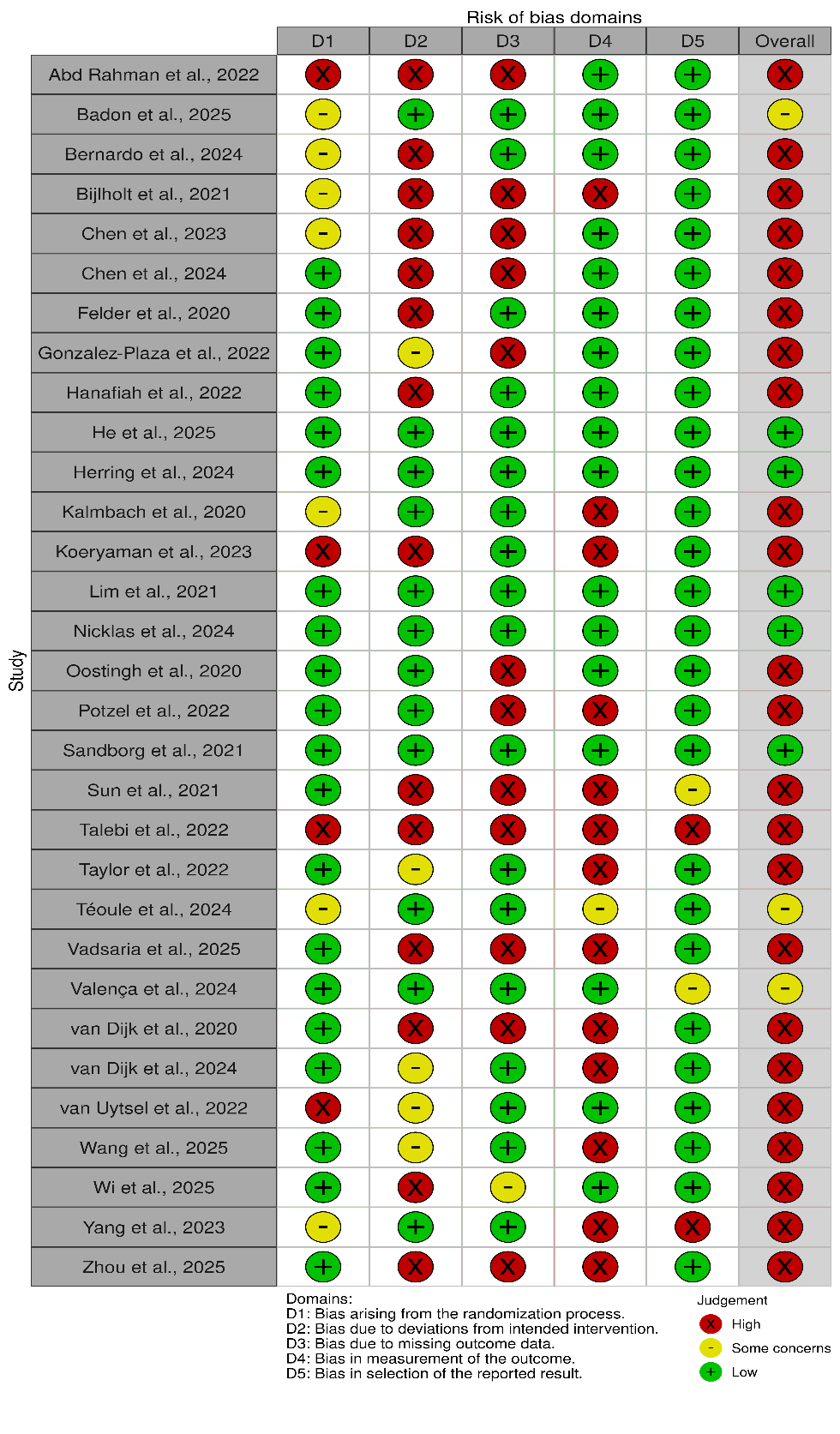


# Supplementary Table 4. Preconception

| Authors | Hanafiah et al. (2022) | van Dijk et al. (2020) | Oostingh et al. (2020) |
| --- | --- | --- | --- |
| Country | Malaysia | Netherlands | Netherlands |
| Setting | 5 health clinics | 1 academic hospital, 4 teaching hospitals, 4 midwifery practices, and several daycare, and child health centers | 6 IVF centers |
| Sample size (n), Intervention | 305 (145) | 254 (women; 218 (109)) | 848 (women; IG: 308, CG: 318, men; IG: 106, CG: 116) |
| Age (years) | IG; 29.1 (4.3), CG; 27.4 (4.1) | 30.6 (5.3) | Women; IG: 33 (29 - 37), CG: 33 (30  - 36); Men; IG: 35 (31 - 39), CG; 35 (31 - 41) |
| Anthropometrics (^a.^ weight, kg, mean, ^b.^ BMI, m/kg2, mean, ^c.^ WC, cm, mean). | ^a.^ IG; 65.1(19.0), CG; 63.8 (17.4), ^b.^IG; 26.9 (7.5), CG; 26.3 (6.8), ^c.^ IG; 81.4 (13.6), CG; 80.7 (13.5). | ^b.^ IG; 24.2 (6.0), CG; 23.7 (5.4) | ^b.^ IG; *Women:* 23.7 (21.6  - 26.7) [Underweight (<20): 35 (11.4), Normal (≥20 to 25): 165 (53.6),  Overweight (≥25 to 30): 68 (22.1), Obese (≥30): 40 (13.0)], *Men:* 25.1 (22.7  - 26.9) [Underweight (<20): 4 (3.8), Normal (≥20 to 25): 46 (43.4), Overweight (≥25 to 30): 45 (42.5), Obese (≥30): 11 (10.4)], CG; *Women:* 23.8 (21.6  - 26.3) [Underweight (<20): 31 (9.7), Normal (≥20 to 25): 172 (54.1),  Overweight (≥25 to 30): 81 (25.5), Obese (≥30): 34 (10.7)], *Men;* BMI: 25.2 (23.2–28.3) [Underweight (<20): 4 (3.4), Normal (≥20 to 25): 50 (43.1),  Overweight (≥25 to 30): 49 (42.2), Obese (≥30): 13 (11.2)]. |
| Intervention | **e-health tool (Jom app):** for young couples; 48 CHPs deliver BCC, peer support, lifestyle challenges (healthy food; avoid oily food, drinking plain water/non-sweetened beverages and avoiding soft drinks; PA; brisk walking, use of stairs instead of the elevator, and planks), and 6 contact points (3 face-to-face, 3 phone) with CHPs over 33 weeks.; CG: standard care, no CHP or app access. Outcome assessment; WC: Standardized WHO measurement; | **mHealth app (Smarter Pregnancy app):** for women contemplating pregnancy or < 13 weeks pregnant. Two versions: full version with personalized coaching for dietary intake (IG; vegetables, fruits, folic acid) via tailored communication (max 3/week) and modified version with limited functionality (CG); DRS assesses intake (0 (healthy) to 9 (unhealthy)); online progress (questionnaires) in IG; monitored at 6, 12, 18, and 24 weeks. | **Web-based program (Smarter Pregnancy app):** for women undergoing IVF/ICSI, with (optional) male partners. IG; tailored coaching (max 3 emails/texts/week) addressing inadequacies, monitored via online questionnaires at 6, 12, 18, and 24 weeks; CG; "light" version, no feedback; subset blood samples for folate validation; outcomes assessed by DRS (24 weeks) for nutrition, LRS for lifestyle (smoking: no smoking (score 0) and daily smoking of 1  - 5 (score 1), 6  - 14 (score 3), or ≥ 15 (score 6) cigarettes/alcohol: no alcohol use (score 0) and 1 - 7 (score 1), 8  - 14 (score 2), or ≥ 15 (score 3), alcoholic beverages (glasses)/week); blood samples collected for folate validation; follow-up questionnaires sent at 36 and 52 weeks for behavior change and pregnancy status. |
| Duration of intervention | 33 weeks | 24 weeks | 24 weeks |
| Outcomes | *Nutrition, rice portion;* reduced in IG (71.7%) vs CG (50%), p = 0.001; other food intakes n.s. ((vegetable and fruit intake (mean, SD), noodles and bread portion size, fried foods, fast food, carbonated drinks, pastries (n, %), sweet local delicacies (‘kuih’) frequency (n, %).  Outcome measure; Food Frequency Questionnaire (FFQ) adapted from a locally validated version. In person  *PA, vigorous job-related activity;* higher in IG (259.9 MET/week) vs CG (153.8 MET/week), p = 0.032; other types of activity not significant. In person  Outcome measure; IPAQ. In person.  *Anthropometrics; Change in weight favored IG*; (-0.83 kg) vs CG (-1,6 kg), p = 0.025; *Change in BMI;* favored IG (-0.33) vs IG (-0.66), p = 0.019; *Obese IG;* showed less weight gain (0.1 kg) vs CG (1.7 kg), p = 0.023. WC; n.s.  Outcome measure; Weight: Standardized site measurement; BMI: Calculated from weight/height, Asian-population WHO cut-offs; Waist-to-Height Ratio: Calculated from waist/height; Waist-to-Hip Ratio: Calculated from waist/hip circumference. In-person  *Metabolic Outcomes;* SBP; IG; 107.6 (SD: 14.0), CG; 104.7 (SD: 10.6), p = 0.031; DBP;  n.s., Elevated BP; p = 0.081, HbA1C; n.s., TC; n.s., HDL; p = 0.632, TGs; n.s.  Outcome measure; HbA1c, TC, HDL, TG: From 10ml fasting blood sample; Lipid Profile: From 10ml fasting blood sample; BP: Standardized measurement. In-person. | *Nutrition, DRS (women);* Reduction in IG was significantly larger than CG (β = 0.75, 95% CI 0.18  - 1.34), mainly due to increased vegetable intake (β = 0.55, 95% CI 0.25 - 0.86).  Outcome measure; DRS via online questionnaire (0 to 9 for women and 0 to 6 for men). Higher scores indicate poorer nutritional and lifestyle habits. | *Nutrition, DRS;* 24 weeks; IG DRS reduction significantly greater vs CG (β = 0.779 women; β = 0.826 men). 36 weeks; IG exhibited a more pronounced decrease from baseline vs CG (β = 0.816 women; β = 0.639 men);  n.s. for folic acid.  Outcome measure; DRS via online questionnaire (0 to 9 for women and 0 to 6 for men). Higher scores indicate poorer nutritional and lifestyle habits.  *Smoking and Alcohol, LRS;*24 weeks; Women: decrease of the LRS in the IG significantly larger than in the CG (β = 0.108, 95% CI 0.021–0.203); n.s. at 36 weeks.  Outcome measure; Lifestyle Risk Score (LRS) calculated from weighted smoking (0-6) and alcohol (0-3) scores. Higher scores indicate poorer nutritional and lifestyle habits. Online questionnaire.  Fertility (Pregnancy Rate); n.s. at 52 wks.  Outcome measure; Online questionnaire. |
| *BCC; Behaviour Change Communication, Wks; Weeks, SBP; Systolic Blood Pressure, DBP; Diastolic Blood Pressure, BP; Blood Pressure, HbA1c; Glycated haemoglobin, TC; Total cholesterol, HDL; High-density lipoprotein, TG; Triglycerides, WC; Waist Circumference, DRS; Dietary Risk Score, LRS; Lifestyle Risk Score, IPAQ; International Physical Activity Questionnaire.* | | | |

# Supplementary Table 5. Pregnancy

| Authors | Abd Rahman et al. (2022) | Chen et al. (2023) | Koeryaman et al. (2023) | Sandborg et al. (2021) | Vadsaria et al. (2025) | Valença et al. (2024) | van Dijk et al. (2020) | Zhou et al. (2025) | Talebi et al. (2022) |
| --- | --- | --- | --- | --- | --- | --- | --- | --- | --- |
| Country | Malaysia | Taiwan | Indonesia | Sweden | Pakistan | Brazil | Netherlands | Iran | Iran |
| Setting (recruitment) | 2 prenatal clinics | 2 prenatal clinics | 11 public maternal and child clinics | Maternity clinics | Antenatal clinics of Aga Khan University Hospital | Diabetes and Pregnancy Outpatient Clinic of Hospital, and Paulista School of Medicina/Federal University | 1 academic hospital, 4 teaching hospitals, 4 midwifery practices, and several daycare, and child health centers | Tertiary maternity hospital | Urmia health centers |
| Sample size (n), IG | 120 (60) | 92 (46) | 112 (56) | 305 (152) | 306 (153) | 81 (47) | 254 (Women; 218 (109)) | 302 (150) | 90 (45) |
| Week of gestation (recruitment) | 13-24 | < 17 | 22-26 | 13 | 1st trimester | 24-34 | < 13 | ≤ 14 | < 20 |
| Age (years) | 28.5 (5.0) | IG; ≤ 35y: 31(67.4%), CG; ≤ 35y: 35(76.1%) | 22.7 | 31.3 (4.1) | IG; 28.7 (4.3), CG; 28.1 (4.1) | IG; 33.4 (6.1), CG; 35.5 (4.8) | 30.6 (5.3) | 29.2 | IG; 29.5 (5.78), CG; 28.9 (5.3) |
| Anthropometrics (a. weight, kg, mean, b. BMI, m/kg2, mean, c. WC, cm, mean) (baseline) | Not mentioned | ^b.^ IG; OW (≥25, <30): 30(65.2%), OB (≥30): 16(34.8%), CG; OW (≥25, <30): 35(76.1%), OB (≥30): 11(23.9%) | ^a.^ IG; 54.75kg (10.9), CG; 54.68kg (8.7) | IG: ^a.^ 68.3 (12.8), ^b.^ 24.7 (4.3), CG; ^a.^ 67.0 (10.2), ^b.^ 23.8 (3.2) | IG; ^b.^ OW or OB (≥23): 98 (64.1), NW (18.5-22.9): 45 (29.4), UW (<18.5): 10 (6.5), CG; OW or OB: 94 (61.4), NW; 45 (29.4), UW; 14 (9.2) | ^b.^ IG; 28.8 (5), CG; 31.1 (7.9) | ^b.^ IG; 24.2 (6.0), CG; 23.7 (5.4) | ^b.^ IG; 21.16 ±2.6, CG; 21.25 ±2.5 | ^b.^ IG; 27.17 ± 4.37, CG; 27.02 ± 4.66 |
| Intervention | **Program through a mobile messaging application (MyPinkMom):** for pregnant women with anemia. IG; daily video dissemination for the first week (6 total), Video 4-6 nutrition-related) and weekly reminders from weeks 2-5. Videos via WhatsApp, each lasting 3-5 min; CG; Standard treatment. Both groups received routine antenatal care); folate and iron supplements, and were prohibited from taking additional medications or vitamins not prescribed by their doctors). | **mHealth MyHealthyWeight (MHW) app + Wearable (wrist-worn Mi Band 5 for fitness tracking):** IG; encouraged to wear the Mi Band 5 for at least 16 hours/day, with a PA target of walking 8500 steps per day; tracking of weight gain, daily diet, and PA, with visual feedback on progress; CG; Standard maternal care. | **App assessed through website (SISFORNUTRIMIL application):** IG; standard prenatal care + SISFORNUTRIMIL application, Indonesian-user tailored (select and enter food consumed in a food record tool, track daily food and drink records, manage portion sizes, and monitor calorie intake without manual counting), logged food records thrice weekly for 12 weeks, once weekly What’s app reminders; CG; Standard prenatal care (nutrition counselling, information sheets, guidelines, and paper-based food records); recorded food intake for 12 weeks post-baseline. All participants received face-to-face baseline measurement and 3 follow-up measurements. | **mHealth (HealthyMoms):** IG; standard maternity care + HealthyMoms app (promote recommended GWG through tracking of healthy diet/recipes and PA/ aerobic and resistance training), ab libitum usage; graphical visualization of progress, traffic light for compliance with recommendations, weight gain chart; CG; standard maternity care, including health monitoring and optional lifestyle education lectures. | **mHealth (PurUmeed Aaghaz):** IG; time-point self-assessments, personalized, behavior-based supplement advice, counseling through the mHealth app during each follow-up supplemented by up to 3 push messages per week, supplement history questionnaire, algorithm, recommendations, continuous monitoring via web portal. WHO guidelines for daily intake: folic acid (0.4 mg), iron (30-60 mg), calcium (1500-2000 mg), and vitamin D3 (200 IU); CG; Face-to-face counselling, no app access, completed paperless questionnaires and received CSUS ranged; 0 (daily use) to 12 (highly inadequate), with scores of 1.5 for 4 to 6 days and 3 for 0 to 3 days of intake. | **Social network (What's app)**: Intervention Group (IG): for women with GDM; IG; outpatient guidance and 6 daily reminders with nutritional tips via WhatsApp at mealtimes. Messages were sent individually and covered the same topics daily without requiring responses; CG; Standard care without additional reminders. | **mHealth app (Smarter Pregnancy app):** for women contemplating pregnancy or < 13 weeks pregnant. Two versions: full version with personalized coaching for dietary intake (IG; vegetables, fruits, folic acid) via tailored communication (max 3/week) and modified version with limited functionality (CG); DRS assesses intake (0 (healthy) to 9 (unhealthy)); online progress (questionnaires) in IG; monitored at 6, 12, 18, and 24 weeks. | **mHealth app (Pregnancy Assistant app) + Social media (WeChat):** IG; Health intervention materials, developed under 7 psychological cognition dimensions based on GWG management research, included 24 items delivered weekly via the "Pregnancy Assistant" app. Regular reminders were sent through the app and WeChat to enhance compliance. Lectures every 2 weeks for 45 minutes, addressing risks of inappropriate GWG, epidemiological knowledge, and preventive dietary and exercise methods, followed by a 15-minute Q&A session; CG; Routine prenatal care and health education. | **Social media platform (What’s app):** IG; virtual training group for communication and support; received educational content (text, audio, video) on PA and dietary management during pregnancy by midwives; 16 sessions over 8 weeks (2 times/week), with each session lasting approximately 90 minutes; Reminders (2 times/week); CG; Quiet match group. |
| Duration of Intervention | 6 weeks | 20 weeks | 12 weeks | 6 months | 24 weeks | 4-8 weeks, depending on patient’s follow-up duration at clinic. | 24 weeks | 14-37 weeks | 8 weeks |
| Outcomes | Nutrition, (FFQ, in person); 6 weeks: 1. Dietary iron intake (mg/day): IG: 20.55 ± 5.12 vs CG: 15.98 ± 3.6 (p < 0.001), 2. Dietary Vitamin C (mg/day): IG: 244.31 ± 233.87 vs CG: 120.76 ± 126.52 (p < 0.001), 3. Dietary tannin (mg/day): IG: 86.94 ± 153.87 vs CG: 686.1 ± 559.69 (p<0.001), 4. Dietary calcium (mg/day): n.s., 5. Dietary phytate (mg/day): n.s. Anemia, Hemoglobin, Full Blood (in person) Count; 6 weeks: IG: 10.75 ± 0.48g/dL vs CG: 10.08 ± 0.62g/dL (p < 0.001); 12 weeks: IG: 11.48 ± 0.53g/dL vs CG: 10.41 ± 0.44g/dL, (p < 0.001). | Nutrition, (Health-related self efficacy in diet and exercise behavior questionnaire, Self-reported via app); 1st trimester: measured at baseline prior to 17 weeks gestation, 2nd trimester: measured at 24 - 26 weeks, 3rd trimester: measured at 34 - 36 weeks: 1. Self-efficacy of diet: IG - β = -0.05 (SE: 0.02), p = 0.007, CG - β = 0.006 (SE: 0.002), p = 0.777. GWG (electronic medical records and self-recorded data, IOM guidelines, in person); 1. Exceeding Weekly GWG (per IOM guidelines) - First trimester: n.s.; Second trimester: IG: 45.0%, CG: 67.4% (p = 0.039); Third trimester: n.s. 2. GWG Amounts (Obese Women, Third Trimester): Intervention: 3.30 ± 1.44 kg, Control: 8.74 ± 1.64 kg (p = 0.023), Adjusted difference: 5.44 kg.  PA, 2.1.(PPAQ + tracker); Total PA: n.s.; 2.2. Self-efficacy of exercise, Health related self efficacy in diet and exercise behavior questionnaire (5-point Likert scale (5 = absolute confidence; 1 = absolutely no) confidence, highest score 125) : n.s. | Nutrition (3-day food records + MDD-W, via app for IG); 12 weeks: 1. MDD-W score: IG: 7.79 (SD 1.20), CG: 7.02 (SD 1.39), p=0.005 (IG improved; vegetables and fruits (mostly any dark green leafy vegetables and pulses, and vegetables rich in vitamin A) and animal sources (meat, poultry, fish, and eggs) vs CG. 2. Energy(kcal): IG: 2502.46 ± 111.6, CG: 2659.34 ± 113.99, p=0.002, Protein(g): IG: 70.17 ± 8.61, CG: 55.84 ± 6.94, p<0.001, Fat(g): IG: 60.13 ± 6.56, CG: 71.09 ± 8.0, p<0.001, Carbohydrate (g): IG: 304.15 ± 56.65, CG: 406.58 ± 64.42, p=0.02, 3. Micronutrients: Iron(mg): IG: 27.44 ± 6.03, CG: 20.5 ± 4.40, p<0.008, Vitamin C(mg): IG: 77.83 ± 9.24, CG: 55.36 ± 70.12, p=0.03. GWG (standard weight scales, in person); Baseline; IG(kg): 54.75 vs CG: 54.68, Change in 4 weeks; IG: 56.8 vs CG: 57.26, Change in 8 weeks; IG: 58.83 vs CG: 58.83, Change in 12 weeks; n.s. | Nutrition, (Web-based 24-hour recall (Riksmaten FLEX; online; Dietary habits, Swedish Healthy Eating Index (9 components; higher score indicating better compliance with dietary guidelines); 6 months (gestational week 37): 1. Adjusted mean difference in index score = + 0.27 points (95% CI: 0.05 to 0.50), p=0.017. 1.1. Notable dietary change; statistically significant reduction only in red meat intake (p=0.027), n.s. for the rest components (Fruit & vegetables, Fibre, Wholegrain, MUFA, PUFA, Fish & shellfish, Sucrose).  PA, (accelerometer, Actigraph wGT3x-BT + diary, wearable); 6 months (gestational week 37): (Moderate to Vigorous PA / MVPA): adjusted mean difference = -0.76min-day (95% CI: -5.34 to 3.8), p=0.74). GWG (Bod Pod; Air-displacement plethysmography; in person; week 14 to week 37); 1. Change in body weight : n.s. on GWG between groups (–0.20 kg; 95% CI –0.98 to 0.59; p = 0.62 for IG vs the CG, n=305). Effect subgroup w/overweight, obesity: (–1.67 kg; 95% CI –3.26 to –0.09; p = 0.031, n=271). 2. Body fatness: adjusted mean difference = +0.05kg (95% CI: -0.65 to -0.76), p = 0.88, 3. Fat-free mass: adjusted mean difference = -0.09kg (95% CI: -0.46 to 0.28), p = 0.64. Metabolic Outcomes (blood samples, in person): 1. Glycemia (Fasting plasma glucose): adjusted mean difference = + 0.06mmol/L (95% CI: -0.03 to 0.15), p=0.21, 2. Insulin resistance (HOMA-IR): adjusted mean difference = +0.10 (95% CI: -0.13 to 0.34), p=0.39. | Nutrition, DRS; n.s. differences between IG and CG, except: Milk and products (Quantity):  Adequate: IG: 0 (0), CG: 0 (0),  Intermediate: IG: 10 (9.3), CG: 3 (2.4)  Inadequate: IG: 97 (90.7), CG: 122 (97.6).  Anemia, Hemoglobin, biochemical test; 24 weeks: Anemia (Hb < 11 g/dL): Decreased IG: from 29% to 24% (-5%), Increased in CG: from 37% to 59% (+22%), p = 0.03. Metabolic Outcomes (biochemical assessment, 24 weeks); 2. Folate Deficiency (<2.6 ng/mL): 0% in both groups at both baseline and endline, 3. Low Ferritin (<10 ng/mL): n.s., 4. Hypocalcaemia (<8.6 mg/dL): Intervention: unchanged at 12.5%, Non intervention: increased from 16% to 58%, p = 0.053, 5. Vitamin D Deficiency (≤20 ng/mL): n.s.  Milk and Milk Products Consumption: Endline: Intervention (n=107) - Inadequate: 97 (90.7%), CG (n=125) - Inadequate: 122 (97.6%). 2. Micronutrients: Folic Acid: Adequate intake at endline: 97.2% IG vs. 96.8% CG, Improvement: +20% IG, +22.4% CG, Adjusted odds ratio (aOR): n.s. Iron: Adequate intake at endline: 95.3% (IG) vs. 72.8% (CG), Improvement: 11.2x (intervention), 2.1x (CG), aOR: 1.31, 95% CI 0.95–1.81, p = 0.10 (CG), Calcium: Adequate intake at endline: 66.4% (intervention) vs. 89.6% (CG, Improvement: 1.2x (IG), 14.2x (CG), aOR: 0.59, 95% CI 0.44–0.79, p <0.001 (significant, favours CG), Vitamin D: Adequate intake at endline: 80.4% (IG) vs. 55.2% (CG), Improvement: 3x (IG), 1.3x (CG), aOR: 1.88, 95% CI 1.43–2.47, p <0.001.  3. CSUS; Greater reduction IG (β=–.27, 95% CI −0.65 to 0.12) - n.s. GDM (medical records); n.s.  Gestational Hypertension (medical records): n.s. Higher odds of sufficient Ca use CG: aOR 0.59 (95% CI 0.44-0.79). | Nutrition, Food consumption (caloric intake, proteins, lipids, carbohydrates, and fiber); Calories; n.s., Protein; n.s., Carbohydrates; T1: Control 119.8 (105.9-145.6), n=33; Intervention 144.1 (121.1-164.8), n=47; p=0.039, T4: Control 124.3 (99.5–142.9), n=18; Intervention 137.8 (113–162.3), n=35; p=0.036, Τ2, Τ3, Τ5, ΤF; n.s., Lipids; T4: Control 43.3 (31.1–56.7), n=18; Intervention 104 (84–119), n=35; p<0.001, T5: Control 45 (36.3–54.3), n=10; Intervention 93.6 (63–113.6), n=25; p<0.001, TF: Control 43.4 (35.3–55.2), n=33; Intervention 86 (54.9–103.9), n=47; p<0.001, Τ1, Τ2, Τ3; n.s., Fibers; T1: Intervention Group: 20.9 g (18.6–27) vs Control Group: 25.8 g (22.7–29.4), p-value: 0.044. Total Final (TF): Intervention Group: 18.9 g (14.5–22.4) vs Control Group: 23.7 g (17.4–27.5), p-value: 0.02, Τ2, Τ3, Τ4, Τ5; n.s. Metabolic Outcomes: 1. Glycemic Control (capillary blood glucose, examination by doctor): The percentage of women demonstrating adequate glycemic control in the IG was higher than in the CG during the following weeks (n.s. for second and eighth week): Third Week: IG: 66% (31 out of 47) vs CG: 35.5% (11 out of 34), p = 0.027. Fourth Week: IG: 83.3% (35 out of 42) vs CG: 35.7% (10 out of 28), p < 0.001. Fifth Week: IG: 85% (34 out of 40) vs CG: 56.5% (13 out of 23), p = 0.04. Sixth Week: IG: 86.5% (32 out of 37) vs CG: 52.6% (10 out of 19), p = 0.011. Seventh Week: IG: 86.2% (25 out of 29) vs CG: 40% (4 out of 10), p = 0.009. 2. Insulin Use (required insulin therapy); n.s. | Nutrition (DRS (women)); Reduction in IG was significantly larger than CG (β = 0.75, 95% CI 0.18  - 1.34), mainly due to increased vegetable intake (β = 0.55, 95% CI 0.25 - 0.86). | Nutrition (Diet Management), including 4 items, with a score range of 4-20 to achieve appropriate GWG; 3rd trimester (T3): The intervention group showed significantly higher scores (IG: 13.68, CG: 12.64; p = 0.007), T1, T2; n.s.  Anthropometrics; Weight management behavior (Gestational Weight Management Behavior Scale) | PA (PPAQ, MET-min/day); 8 weeks; 1. IG; 175.31 ± 61.54 MET-min/day; CG; 75.75 ± 34.41 MET-min/day, p < 0.001; 2. PA Subgroups: Light (CG: 5.35 ± 3.17, IG: 12.77 ± 4.32), moderate (CG: 1.61 ± 2.58, IG: 8.31 ± 4.88), and severe activities (CG: 0.08 ± 0.29, IG: 0.23 ± 0.35), p<0.05. Sedentary activity: n.s. Other Activity Types (Exercise, Home, Occupational activities): Significant improvements in intervention group in all three categories post-intervention; Exercise: CG: 0.25 ± 0.49, IG: 1.87 ± 0.93, p=< 0.001, Home; CG: 5.45 ± 3.78, IG: 14.40 ± 5.70, p<0.001, Occupational; CG: 0.62 ± 1.46, IG: 1.74 ± 2.70, p=0.03. GWG (Seca scale); 8 weeks (post-intervention); Weight changes: Pre-intervention: CG: 72.17 ± 13.63 kg vs IG: 72.41 ± 13.25 kg. Post-intervention: Control: 79.00 ± 15.31 kg vs Intervention: 74.57 ± 13.43 kg, Significant within-group changes (p < 0.001), but between-group: n.s. |
| OW; Overweight, OB; Obesity, NW; Normal weight, UW: Underweight, PA; Physical Activity, TAU; Treatment As Usual, BCC; Behaviour Change Communication, Wks; Weeks, SBP; Systolic Blood Pressure, DBP; Diastolic Blood Pressure, BP; Blood Pressure, HbA1c; Glycated haemoglobin, TC; Total cholesterol, HDL; High-density lipoprotein, TG; Triglycerides, MDD-W; Minimum Dietary Diversity for Women , CSUS; Cumulative Supplement Use Score, PA; Physical Activity; PPAQ; Pregnancy Physical Activity Questionnaire, IPAQ-SF; International Physical Activity Questionnaire – Short Form, PSQI; MVPA; Moderate to Vigorous Physical Activity; Pittsburgh Sleep Quality Index, ISI; Insomnia Severity Index, TST; Total Sleep Time, SE; Sleep Efficiency, TWT; Total Wake Time, GWG; Gestational Weight Gain, GDM; Gestational Diabetes Mellitus, WC; Waist Circumference. | | | | | | | | | |

# Supplementary Table 6. Postpartum

| **Authors** | Bijlholt et al., (2021) | Lim et al., (2021) | Potzel et al., (2022) | He et al., (2025) | Badon et al., (2025) | Taylor et al., (2022) | Nicklas et al., (2024) | Van Uytsel et al., (2022) | Herring et al., (2024) | Chen et al., (2024) | Kalmbach et al., (2020) | Sun et al., (2021) | van Dijk et al., (2024) | Yang et al., (2023) | Wang et al., (2025) |
| --- | --- | --- | --- | --- | --- | --- | --- | --- | --- | --- | --- | --- | --- | --- | --- |
| **Country** | Belgium | Singapore | Germany | China | USA | Australia | USA | Belgium | USA | Taiwan | USA | China | Netherlands | China | China |
| **Setting** | 6 hospitals | National University Hospital (NUH) | Medical Center of the Ludwig-Maximilians-Universität in Munich (main study site), the German Diabetes Center in Düsseldorf and the Institute for Diabetes Research and Metabolic Diseases in Tübingen | Obstetrics department of a tertiary general hospital (The Second Affiliated Hospital of Fujian Medical University) | Letter or email invitations | 1. media releases and social media posts, and 2. invitation letters emailed to women with previous GDM who were registered on the Diabetes Australia National Diabetes Supply Scheme Register | University of Colorado Hospital on the University of Colorado Anschutz Medical Campus in Aurora, Colorado | 6 hospitals: University Hospital Leuven, University Hospital Antwerp, Gasthuiszusters Hospitals Antwerp, St-Franciscus Hospital in Heusden-Zolder, Jessa Hospital in Hasselt, and Hospital Oost-Limburg in Genk. | WIC clinic waiting rooms | 2 prenatal clinics | 6-hospital healthcare system | Obstetrics clinic of a tertiary hospital | Website advertisement | First-class tertiary general hospital | 2 tertiary hospitals |
| **Sample size (n), Intervention** | 1046 (504) | 189 (96) | 66 (33) | 72 (36) | 99 (50) | 76 (IG; High Personalisation (HP): 25, Medium Personalisation (MP): 23, Low Personalisation (LP): 28) | 81 (53) | 1075 (551) | 300 (149) | 92 (46) | 91 (46) | 168 (84) | 81 (53) | 144 (72) | 160 (80) |
| **Age (years)** | 31.3 (3.9) | 32.5 (4.3 | IG; 37.0 (3.1), CG; 35.7 (4.4) | IG; 30.33 (2.9), CG; 30.23 (3.6) | IG; 31.9 (5.1), CG; 32.7 (4.5) | HP group: 36.0 (5.4), MP group: 34.6 (5.3), LP group: 37.3 (4.2) | 31 (5.4) | IG; 31.2 (4.0), CG; 31.4 (3.9) | 28.3 (4.7) | IG; ≤35y: 31 (67.4%), CG; ≤35y: 35 (76.1%) | 29.03 (4.2) | 29.91 (4.0) | 29.31 (5.0) | 27.08 (3.03) | 30.54 (3.9) |
| **Anthropometrics (^a.^ weight, kg, mean, ^b.^ BMI, m/kg2, mean, ^c.^ WC, cm, mean), d. Gestational Weight Gain (GWG, kg)** | ^b.^ IG; Pre-pregnancy, n(%) - NW: 260 (48); OW 193 (35.6); OB: 89 (16.4), CG; Pre-pregnancy BMI category n(%) - NW: 249 (49.4); OW 181 (35.9); OB: 74 (14.7) | IG; ^a.^ First trimester, mean (SD): 63.1 (16.0), ^b.^ First trimester, median (IQR): 24.6 (20.7-27.8), ^b.^ At delivery, median (IQR): 26.9 (23.0-29.5), ^d.^ Mean (SD): 8.84 (4.2), CG; ^a.^ First trimester, mean (SD): 61.9 (11.3), First trimester, median (IQR): 23.4 (21.0-27.0), At delivery, median (IQR): 25.8 (22.8-28.6), ^d.^ Mean (SD): 9.19 (4.7). | IG; ^a.^ Baseline: 67.6 (61.9–78.2), ^b.^ 26.0 (22.6–28.9), CG; ^a.^ Baseline: 72.5 (66.6-84.5), ^b.^ 27.3 (23.9-30.3) | ^b.^ IG; At delivery: 24.14 (2.4), CG; 23.81 (2.2) | Not mentioned | ^b.^ OW (≥ 25 to ≤30kg/m2): HP group: 14 (56), MP group: 8 (35), LP group: 16 (57), OB (>30 kg/m2): HP group: 11 (44), MP group: 15 (65), LP group: 12 (43) | ^b.^ IG; 32.3 (5.2), CG; 32.4 (4.6) | Not mentioned | IG; ^b.^ 35.9±6.0 kg/m2, Obesity ( ≥ 30 kg/m2): 124 (83.2%), a. 96.2 (18.0), CG; b. 36.0±5.9 kg/m2, OB 131 (86.8%), a. 97.8 (17.1) | ^b.^ IG; OW (≥25, <30): 30(65.2%), OB (≥30): 16(34.8%), CG; OW: 35(76.1%), OB: 11(23.9%) | ^b.^ IG; OB (BMI ≥ 35) (n; %): 9/44; 20.5%, CG; OB (n; %): 3/42; 7.1% | ^b.^ IG; 22.76 (2.9), CG; 22.07 (3.0) | Not mentioned | ^a.^ IG; 50.11 (5.3), CG; 51.80 )6.0) | ^b.^ Prepregnancy; IG; 22.92 (4.5), CG; 22.67 (3.4), Baseline; IG; 23.73 (4.60), CG; 23.12 (3.4) |
| **Intervention** | **mHealth app + activity tracker (Withings GO) + weighing scale (Withings Body+);** structured lifestyle program for women experienced excessive GWG: 4 coaching sessions at intervals of 6 weeks, 8 weeks, 12 weeks, and 6 months postpartum, addressing nutrition, eating behavior, PA, sedentary behavior and mental wellbeing delivered by a combination of face-to-face coaching and purpose-designed smartphone application; CG; not offered four coaching sessions and the smartphone application. | **mHealth (Nutritionist Buddy app);** for women with a recent history of GDM, IG; personalized weight targets, calorie and activity goals, and allowed daily logging of food intake from a database of over 11,000 foods, prompts for healthier food alternatives, step-counting, and access to educational 16 video clips (diet, exercise, emotional health, breastfeeding and weaning diet for babies), allowed live interactions with a multidisciplinary team; CG; Standard care + follow-up appointment at 6 weeks postpartum for routine postnatal check. | **mHealth (TRIANGLE app);** for women with a recent history of GDM (3-18 months), IG; 6-month program designed for iPhone users that focuses on enhancing habits related to mind and emotion, PA, nutrition, and sleep, incorporating personalized challenges and coaching. It utilizes a comprehensive app with features such as individualized challenges, chat-based coaching, and a resource library; CG; lifestyle change information for diabetes prevention. | **mHealth (Keep app);** for women with a recent history of GDM, IG; Received routine postpartum care, installed the Keep app and received training to use features related to PFMT, watched the “Kegel Training” video in the app and completed daily training punch cards, joined a community forum for peer interaction, and performed daily PFMT lying on a bed or floor (15 min, the pelvic floor muscles were continuously contracted for no less than 3 s, relaxed and rested for 2–6 s, and the process was repeated 3 times a day or 150–200 anal contractions); CG; Routine postpartum care. | **e-health (MomZing);** for women with high risk for PPD, IG; received access to an eHealth PA intervention, online library of 98 ten-minute exercise videos, instruction on safe exercises with their baby, without the need for exercise equipment, weekly PA goals and track progress visual; CG; Usual postpartum care. | **e-health (Body Balance Beyond) + Web-based platform (Zoom);** for women with overweight or obesity and a recent history of GDM (between past 5 years); IG; High Personalisation (HP) Group: Access to the "Body Balance Beyond" online program for 3 months, tailored for women with a history of GDM. Included 5 individual video coaching sessions with a dietitian and exercise physiologist. Received personalized feedback, goal-setting tools, and resources for healthy lifestyle behaviors. Weekly text messages providing motivation and support, Video coaching sessions via the Zoom platform. Medium Personalisation (MP) Group: Access to the same online program as the HP group but without video coaching sessions. Received personalized text messages over the 3-month period. Low Personalisation (LP) Group: Access to the "Body Balance Beyond" program website only, without any personalized coaching or text messages; CG; Ν/Α. | **mHealth (FAB app) + Wearable (Fitbit) + Weight scale;** for women with a recent singleton delivery complicated by gestational hypertension, preeclampsia, preterm delivery, delivery of an SGA neonate, and/or gestational diabetes; IG; received a Fitbit (aimed to increase steps by 1,000 each week to reach 10,000 steps/day, and to gradually increase PAy by 10 minutes daily until achieving 45-60 minutes) and a body weight scale, along with the app; daily content focused on diet, PA, and weight tracking; interactive content such as quizzes, PA suggestions, and self-efficacy strategies; earn points for engaging with the app; CG; instructed to download the Text4baby app, which provided 2-4 text messages per week with maternal and child health information; no weight loss-focused intervention. | **mHealth + Activity tracker (Withings Go) + Weighing Scale (Withings Body+);** for women experienced excessive GWG; IG; 4 face-to-face lifestyle coaching sessions conducted at 6 weeks, 8 weeks, 12 weeks, and 6 months postpartum; Each participant was assigned a specific coach for personalized guidance; Session Topics: Sessions 1 & 2: General lifestyle discussions focusing on nutrition, PA, and mental health, Session 3: In-depth coaching on nutrition, eating behavior, and food intake, Session 4: Targeted coaching on PA, sedentary behavior, and mental health (stressors and social support); CG; No intervention. | **mHealth + Web-based platform (Facebook);** for self-reported pre-pregnancy BMI ≥ 25kg/m2 IG; received standard WIC care plus a multi-component postpartum weight loss intervention; Nutrition and PA behavior change goals, Skills training materials sent via text messages and video links, Interactive self-monitoring text messages with personalized feedback, Interpersonal counselling support through health coach calls and Facebook, Prizes; CG; Standard of care provided to postpartum people at WIC. | **mHealth (MyHealthyWeight (MHW) app) + Wearable (Mi Smart Band 5);** for pregnant women with OW or OB and follow-up postpartum (6 months); IG; weekly SMS encouragement to healthy behaviors, e.g., achievements related to weight (IOM guidelines) and PA (8500 steps/day); CG; Standard maternal care. | **Website (Sleepio);** for women with clinically significant insomnia symptoms; IG; granted access until they completed six sessions or gave birth; CBT-I (1. Behavioral Components: Sleep restriction, Stimulus control, 2. Cognitive Components: Cognitive restructuring, Paradoxical intention, 3. Additional Techniques: Progressive muscle relaxation, Sleep hygiene); Minimum time in bed prescribed was not less than 6 hours; CG; Online sleep education, 6 weekly emails. | **mHealth (Spirits Healing app), Social media platform (WeChat);** for women with positive depression symptom screening; IG; program adapted from MBCT Each week had: • Thematic curriculum (text, audio, visual) • Formal training (e.g., body scan, breathing, meditation) • Informal practices (e.g., mindful eating/walking). 8 weekly classes, 2.5 hours each, and daily home practice assignments for around 45 minutes each day; CG; Attention control. | **mHealth (eHealth application including HRV-BF training targeted at smoking cessation and stress reduction);** for women currently smoking; IG; modules including a personalized stop-smoking plan, smoking diary, HRV-biofeedback training, psychoeducation, and stress management exercises; CG; Active control group. | **Social media (WeChat online service);** IG; divided into 6 subgroups, each subgroup engaged in continuous group care through WeChat online groups (6 sessions were held from 30 to 38 weeks of gestation; discussions, health education via various methods (PPT, role-playing), and personal sharing among participants); Intrapartum period; standard care, participants received online peer support from midwives through WeChat during labor; CG; Prenatal: routine examinations, fetal monitoring, health education; Intrapartum: entered delivery room alone due to COVID-19, family support via calls, midwives provided pain relief guidance. | **Social media (WeChat mini program “Mom Sleep Well”);** for women with subthreshold to clinical insomnia symptoms; IG; digital mindfulness-based intervention for prenatal insomnia consists of six weekly modules, each featuring a video-based thematic course and daily audio-based formal mindfulness practices. Participants learn strategies for managing insomnia, including mindfulness techniques and sleep hygiene, while engaging in both formal and informal mindfulness practices. The modules are accessed through a WeChat miniprogram; CG; Standardized care (TAU). |
| **Duration of intervention** | ~20 weeks, from 6 weeks postpartum to 6 months postpartum | 6 weeks postpartum and 4 months postpartum | 6 months | 4 weeks (from postpartum week 2 to week 6) | 3- and 6 months | 3 months | 12 weeks | ~20 weeks, from 6 weeks postpartum to 6 months postpartum | 12 months | 20 weeks (follow-up at 6 months postpartum) | Access to the Sleepio program until they either: Completed 6 sessions of digital CBTI, or Gave birth (follow-up at 6 weeks after childbirth). | T3; 8 weeks after allocation (endpoint of the intervention), T5; 6 weeks after delivery. | T2; 2 weeks after birth, T3; 3 months after birth | Not mentioned. At 42 days postpartum. | Not mentioned. At 42 days postpartum. |
| **Outcomes** | • Nutrition; Restrained Eating (Three Factor Eating Questionnaire Revised 18-item version): o At the end of intervention (6 months postpartum), IG scored 1 point higher (Mean Difference 1.0, 95% CI 0.5 to 1.5; p < 0.001). o At 6-month follow-up, IG maintained a 1.3 points higher score in the normal weight stratum (p = 0.001). • Uncontrolled Eating (Three Factor Eating Questionnaire Revised 18-item version): o At 6 months postpartum, IG scored 1 point lower (Mean Difference −1.0, 95% CI −1.9 to −0.2; p = 0.02). o At 6-month follow-up, IG had 2.2 points higher scores in the overweight stratum (p = 0.006). • Energy Intake (kcal) (Food Frequency Questionnaire): o At 6 months postpartum, energy intake was 69 kcal lower in IG (Mean Difference −69, 95% CI −123 to −15; p = 0.01). o At 6-month follow-up, energy intake remained 138 kcal lower in the obese stratum of IG (p = 0.09, n.s.). • PA (International Physical Activity Questionnaire); o At 6 month follow-up, women with overweight in IG had borderline non-significantly higher PA compared to the control group (p = 0.053). o No significant differences were found in sedentary time between groups at any measurement points. | 1. Nutrition (all p<0.001);  a. Nutritional Intake (at 6 weeks, 3-day food diary): i. Total Caloric Intake: Reduced by 591.3 kcal (95% CI −717.1 to −465.4) ii. Total Fat: Reduced by 25.3 g (95% CI −31.7 to −19.0) iii. Protein: Reduced by 24.1 g (95% CI −31.1 to −17.0) iv. Carbohydrates: Reduced by 67.4 g (95% CI −84.6 to −50.2) v. Sugar: Reduced by 22.9 g (95% CI −30.0 to −15.7) b. Nutritional Intake (at 4 months, 3-day food diary): i. Total Caloric Intake: Reduced by 614.2 kcal (95% CI −751.5 to −476.9) ii. Total Fat: Reduced by 20.7 g (95% CI −27.2 to −14.2) iii. Protein: Reduced by 25.7 g (95% CI −33.1 to −18.3) iv. Carbohydrates: Reduced by 71.2 g (95% CI −90.3 to −52.1) v. Sugar: Reduced by 27.9 g (95% CI −35.7 to −20.1) 2. PA (step-counting, app + RAND-12 questionnaire); IG: logged a mean step count of 4065 steps at 6 weeks, which increased to 4880 steps at 4 months (p=0.04). 3. Postpartum Weight Retention/Loss (kg) (4 month postpartum); Absolute difference in weight (kg) from first trimester: Adjusted: IG: −0.63, CG; 0.08, p=0.18. 4. WC (cm, 4 month postpartum): Adjusted: IG: 84.1, CG: 85.6, p=0.34.  5. Systolic Blood Pressure (SBP, mmHg, 4 months postpartum); Adjusted: IG: 107.0, C G:106.7, p=0.86. 6. Right hand grip strength (kg, 4 month postpartum); Adjusted: IG: 22.40, CG: 22.84, p=0.52.  7. Exclusive breastfeeding (days, 4 month postpartum); Adjusted: IG: 50.5, CG: 48.3, p=0.68. 8. Fasting OGTTb test (mmol/L, 4 month postpartum); Adjusted: IG: 4.73, CG: 4.74, p=0.73. 9. 2-hour OGTT (mmol/L, 4 month postpartum); Adjusted: IG: 6.63, CG: 6.58, p=0.83. | 1. Single components of DPP (per-protocol);  a. Nutrition; Fiber Intake: IG had higher fiber intake at V2 compared to CG (p=0.007). b. Nutrition; Fat intake at V2 [% of total kcal]: p=0.78.  c. Nutrition; Saturated fat intake at V2 [% of total kcal]: p=0.10. d. PA at V2 (IPAQ, ergospirometry, Garmin vívosmart HR® fitness tracker) [min/week]: p=0.97.  e. Δ body weight V1 to V2 [% of baseline]: 1. BMI ≥23: p=0.22, 2. BMI <23: p=0.42. 2. Metabolic Outcomes:  HbA1c (IG: 5.31, CG: 5.26, Difference: 0.05, p-value: 0.180), C-peptide (IG: 383.3, CG: 396.6, Difference: 0.97, p-value: 0.642), HOMA-IR (IG: 1.11, CG: 1.06, Difference: 0.95, p-value: 0.660), Total cholesterol (IG: 5.17, CG: 5.22, Difference: -0.05, p-value: 0.686), HDL (IG: 1.61, CG: 1.61, Difference: -0.001, p-value: 0.991), LDL (IG: 3.15, CG: 3.15, Difference: 0.001, p-value: 0.996), ALT (IG: 19.92, CG: 21.55, Difference: 0.92, p-value: 0.227), AST (IG: 21.64, CG: 22.36, Difference: 0.97, p-value: 0.362), hsCRP (IG: 2.29, CG: 1.92, Difference: 1.19, p-value: 0.251), Interleukin-6 (IG: 1.28, CG: 1.20, Difference: 1.08, p-value: 0.446) (-0.3–0.7) (p=0.50). | 1. PA (after 4-week intervention): 1. Maternal pelvic floor muscle strength: The maternal pelvic floor class I and II muscle strengths in the test group were significantly higher than those in the control group (p < 0.001). 2. PFMT adherence scores: Post-Intervention: Both groups showed significant increases in scores, with the experimental group scoring higher than the control group (p < 0.001). 2. Urinary Incontinence (UI, International Consultation on Incontinence Questionnaire Short Form (ICIQ-SF): Post-Intervention: Both groups showed decreased scores, with the experimental group having a significantly lower score than the control group (p < 0.001). | 1. PA (Pregnancy Physical Activity Questionnaire for sr- and ActiGraph GT3X+ accelerometer for dm-MVPA);  a. 3 Months Post-randomization;  i. Self-Reported Moderate or Vigorous PA (sr-MVPA, Pregnancy Physical Activity Questionnaire): Control: 12.7 (SD: 14.7), Intervention: 15 (SD: 15), Adjusted Mean Difference: 3.3 (95% CI: -3.3 to 10) ii. sr-MVPA-MomZing: Control: 2.2 (SD: 3.2), Intervention: 5.6 (SD: 7), Adjusted Mean Difference: 2.8 (95% CI: 0.2 to 5.4) b. 6 Months Post-randomization i. Device-Measured Moderate or Vigorous PA (dm-MVPA, ActiGraph GT3X+ accelerometer): Control: 62.3 (SD: 53.8), Intervention: 58.9 (SD: 45.5), Adjusted Mean Difference: 2.5 (95% CI: -21.1 to 26.1) ii. Self-Reported Moderate or Vigorous PA (sr-MVPA, Pregnancy Physical Activity Questionnaire): Control: 16.3 (SD: 16.6), Intervention: 11.2 (SD: 12.3), Adjusted Mean Difference: -4.9 (95% CI: -12.6 to 2.8) c. sr-MVPA-MomZing: Control: 2.5 (SD: 4.6), Intervention: 2.7 (SD: 3.8), Adjusted Mean Difference: -0.6 (95% CI: -3 to 1.8) 2. Sleep (PHQI); Sleep quality (at 3 months, ΙΤΤ): CG: 10.1 (4.2) vs IG: 9.6 (4), adjusted mean difference (95% CI): 0.5 (−1.2 to 2.1). Sleep quality (at 6 months, ΙΤΤ): CG: 8.7 (3.9) vs IG: 8.7 (3.4), adjusted mean difference (95% CI): 0.5 (−1 to 2.3). | 1. Nutrition (3 months): Diet Quality (adherence to nutrient-dense food recommendations based on the Australian Dietary Guidelines, Australian Recommended Food Score (ARFS)) (max. 73): a. HP Group: -0.89 (95% CI: -1.37, -0.42) b. MP Group: 1.09 (95% CI: 0.53, 1.64) c. LP Group: -0.31 (95% CI: -1.4, 0.8), P-value: 0.73 2. PA (3 months, Godin Leisure-time Exercise Questionnaire): Moderate to Vigorous PA (MVPA) (minutes/week): a. HP Group: 26.77 (95% CI: 23.52, 30.02) b. MP Group: -23.97 (95% CI: -31.41, -16.52) c. LP Group: 64.70 (95% CI: 52.18, 77.21), P-value: 0.08 3. Weight (3 months, self-reported): Weight (kg): a. HP Group: -1.99 (95% CI: -2.00, -1.97) b. MP Group: -1.93 (95% CI: -1.97, -1.89) c. LP Group: -1.27 (95% CI: -1.33, -1.21), p-value: 0.660 (no significant group-by-time effects) | 1. Nutrition (food frequency questionnaire (2005 Block FFQ)); Dietary Changes (over-time changes):  • Caloric Intake: Intervention Group Month 6: Decrease of -718.2 kcal (p = 0.0004) and Intervention Group Month 12: Decrease of -784.1 kcal (p = 0.0002). CG: n.s. • % of Kcal from Carbohydrates: -2.9 (-5.4 to -0.3) (p = 0.0310) -6.5 (-9.2 to -3.8) (p < 0.0001). • % of Kcal from Sweets: Intervention Group Month 6: -5.1 (-7.7 to -2.5) (p = 0.0002), Intervention Group Month 12: Decrease of -6.7% (p < 0.0001).  • Glycemic load (% Change): Intervention Group Month 6: -35.9 (-46.7 to -23.0) (p = < .0001), : Intervention Group Month 12: -44.2 (-54.0 to -32.5) (p = < .0001).  • Vegetable intake, Fruit intake, Whole grains, Fiber, Saturated Fat: n.s. 2. PA Changes (Pregnancy Physical Activity Questionnaire (PPAQ), Month 12): • Sedentary Hours per Week: Both groups showed significant reductions, with the intervention group reducing sedentary hours by -19.6 hours (p < 0.0001). 3. Metabolic Outcomes: Cardiometabolic Risk Measures:  • LDL Cholesterol: Intervention Group Month 6: Mean decrease = -15.6 mg/dL (p < 0.0001), Intervention Group Month 12: Mean decrease = -14.7 mg/dL (p = 0.0001), Mean difference between groups: n.s. • Adiponectin: Intervention Group Month 12: Mean increase = 2.5 µg/mL (p = 0.0012), Mean difference between groups: n.s. • HOMA-IR: Mean difference between groups: n.s. • Systolic Blood Pressure (mmHg): Mean difference between groups: n.s. • Diastolic Blood Pressure (mmHg): Mean difference between groups: n.s. • Fasting Triglycerides* (% change): Mean difference between groups: n.s. • HDL Cholesterol: Mean difference between groups: n.s. • Fasting Glucose* (% change): Mean difference between groups: n.s. 4. Weight (all participants):  • Weight (kg) i. Baseline (Mean ± SD): Control: 87.5 (15.6), Intervention: 87.5 (14.6) ii. Change at 6 months (Mean change, 95% CI): Control: −0.5 (−2.5 to 1.4), p = 0.60, Intervention: −1.6 (−3.1 to −0.1), p = 0.0359, Difference between groups: −1.1 (−3.5 to 1.4), p = 0.40 iii. Change at 12 months (Mean change, 95% CI): Control: −1.8 (−3.8 to 0.3), p = 0.09, Intervention: −2.8 (−4.2 to −1.4), p = 0.0002, Difference between groups: −1.0 (−3.5 to 1.5), p = 0.42 • BMI (kg/m²) i. Baseline (Mean ± SD): Control: 32.4 (4.6), Intervention: 32.3 (5.2) ii. Change at 6 months (Mean change, 95% CI): Control: −0.1 (−0.8 to 0.6), p = 0.79, Intervention: −0.5 (−1.1 to 0.0), p = 0.0589, Difference between groups: −0.4 (−1.3 to 0.5), p = 0.37 iii. Change at 12 months (Mean change, 95% CI): Control: −0.5 (−1.3 to 0.3), p = 0.23, Intervention: −1.3 (−1.8 to −0.7), p < 0.0001, Difference between groups: −0.8 (−1.7 to 0.2), p = 0.13 • Weight change from pre-pregnancy (kg): i. At 6 months (Mean change, 95% CI): Control: +2.9 (0.2 to 5.6), p = 0.0342, Intervention: +3.8 (1.8 to 5.8), p = 0.0002, Difference between groups: 0.9 (−2.4 to 4.3), p = 0.58 ii. At 12 months (Mean change, 95% CI): Control: +1.5 (−1.3 to 4.3), p = 0.30, Intervention: +2.5 (0.6 to 4.5), p = 0.0115, Difference between groups: 1.1 (−2.3 to 4.4), p = 0.54.  • WC (cm): Mean difference between groups: n.s. | 1. Nutrition; From 6 weeks to 6 months postpartum, participants who improved nutrition-related lifestyle behaviors demonstrated significantly better anthropometric outcomes compared to those who did not improve. Specifically, women who reported improved energy intake (24-item Food Frequency Questionnaire) achieved greater weight loss (−3.1 ± 4.2 kg vs. −2.2 ± 3.8 kg, p = 0.05), fat percentage reduction (−2.6 ± 2.9% vs. −1.4 ± 2.7%, p = 0.01), and WC decrease (−5.6 ± 6.5 cm vs. −4.1 ± 5.5 cm, p = 0.01). Improvements in restrained eating (Three-Factor Eating Questionnaire Revised 18-item version (TFEQ-R18)) were associated with a greater reduction in fat percentage (−2.2 ± 3.0% vs. −1.4 ± 2.6%, p = 0.02), but not with significant changes in weight or WC. In contrast, improvements in uncontrolled eating (Three-Factor Eating Questionnaire Revised 18-item version (TFEQ-R18)) showed the strongest associations, with greater weight loss (−3.5 ± 4.2 kg vs. −2.5 ± 3.7 kg, p < 0.001), fat percentage reduction (−3.1 ± 2.9% vs. −1.9 ± 2.8%, p = 0.005), and WC decrease (−5.3 ± 6.0 cm vs. −3.6 ± 5.0 cm, p = 0.002). In contrast, emotional eating improvements (Three-Factor Eating Questionnaire Revised 18-item version (TFEQ-R18)) were not associated with significant differences in weight loss, fat percentage, or WC. 2. PA (Physical Activity Questionnaire (IPAQ)); From 6 weeks to 6 months postpartum, improvements in PA were not associated with significant overall changes in weight (−2.9 ± 4.3 vs. −2.5 ± 3.9 kg, p = 0.42), fat percentage (−2.1 ± 2.9% vs. −1.7 ± 2.7%, p = 0.25), or WC (−4.3 ± 5.2 vs. −3.8 ± 4.4 cm, p = 0.41). A significant effect was observed only in women with obesity, who showed a greater waist reduction when PA improved (−5.7 ± 6.3 vs. −1.5 ± 5.5 cm, p = 0.01). For sedentary behavior, no significant differences were found in weight (−2.6 ± 3.7 vs. −2.8 ± 3.9 kg, p = 0.55), fat percentage (−1.9 ± 2.9% vs. −1.5 ± 2.8%, p = 0.19), or WC (−3.5 ± 6.1 vs. −3.1 ± 4.5 cm, p = 0.84). 3. Postpartum weight retention (BIA): n.s. from baseline (6 weeks postpartum) until the end of the postpartum intervention at 6 months postpartum  4. Fat percentage (BIA, Tanita): n.s. from baseline (6 weeks postpartum) until the end of the postpartum intervention at 6 months postpartum  5. WC change (Seca 201 measuring tape): n.s. from baseline (6 weeks postpartum) until the end of the postpartum intervention at 6 months postpartum | 1. Weight Change Outcomes (SECA digital scales + BodyTrace scales);  o 6-Month Adjusted Mean Weight Change: Intervention: 1.7 kg, Usual Care: 1.8 kg, Difference: −0.1 kg, p-value: 0.9 o 12-Month Adjusted Mean Weight Change: Intervention: 1.1 kg, Usual Care: 1.6 kg, Difference: −0.6 kg, p-value: 0.5 o Weight Change by Engagement Level at 12 Months: High Engagement: −0.6 kg, Low Engagement: 2.4 kg, Difference: −3.0 kg, p-value: 0.01 2. Metabolic Outcomes; Cardiometabolic Outcomes: o Systolic Blood Pressure Change at 6 Months (GE Dinamap ProCare 400 Vital Signs Monitor): Intervention: −1.7 mmHg, Usual Care: 2.1 mmHg, Difference: −3.8 mmHg, p-value: 0.03 o Systolic Blood Pressure Change at 12 Months (GE Dinamap ProCare 400 Vital Signs Monitor): Intervention: −1.6 mmHg, Usual Care: 2.4 mmHg, Difference: −4.0 mmHg, p-value: 0.02 o Systolic Blood Pressure Change for Patients with BP ≥130/80 mmHg at Baseline (GE Dinamap ProCare 400 Vital Signs Monitor): Intervention: −10.4 mmHg, Usual Care: 1.1 mmHg, Difference: −11.5 mmHg, p-value: <0.0001 o Other Cardiometabolic Outcomes (No Significant Differences): 1. Diastolic Blood Pressure (GE Dinamap ProCare 400 Vital Signs Monitor): p-value = 0.30 2. Total Cholesterol (Fasting blood samples): p-value = 0.87 3. LDL Cholesterol (Fasting blood samples): p-value = 0.61 4. HDL Cholesterol (Fasting blood samples): p-value = 0.75 5. Hemoglobin A1c (Fasting blood samples): p-value = 0.16 | 1. Maternal Weight Outcomes: At 6 months postpartum, the mean body weight for the Intervention Group (IG) was 72.55 kg, while the Control Group (CG) had a mean weight of 72.58 kg (p = 0.982). The differences in mean body weight at this time point were not statistically significant. 2. Postpartum Weight Retention was also similar between groups, with the IG retaining −1.52 kg and the CG retaining −1.48 kg (p = 0.982). | 1. Sleep (PSQI, 6 weeks); 1. Sleep quality; PSQI scores did not differ between treatment conditions [F(1,84) = 0.27, p = 0.61]. 2. Sleep duration; CBTI patients slept significantly longer than controls after childbirth by 40 min per night [6 h 6 min vs 5 h 26 min; F(1,84) = 6.50, p = 0.01]. 2. Insomnia (ISI, 6 weeks); Insomnia Symptoms: At the 6-week postnatal follow-up, no group difference in ISI scores or sleep onset insomnia; CBTI patients had lower rates of sleep maintenance insomnia than controls (PSQI, 65.9% vs 85.4%, p = 0.04). 3. Nocturnal Cognitive Arousal: A main effect of time was observed, with presleep arousal scale cognitive factor (PSAS-C) scores decreasing by 7.99 points from pretreatment to postnatal follow-up (F(1,84) = 4.09, p = 0.046). However, no Treatment X Time effect was observed. | 1. Sleep (PSQI, T5); Sleep-related problems; No Significant Effects. ATG: 7.59 (3.768) –0.31, p=0.14, MTPG: 8.12 (3.585), 0.29, p=0.13, Between-group difference (Cohen d): –0.14, –0.56 to 0.27. | 1. Smoking (T2, T3, online questionnaires):  a. Smoking cessation (yes/no): i. T2: Significant increase compared to T1 (OR = 1.61, 95% CI [1.02–2.56], p = .042). ii. T3: No further increase; cessation rates stabilized (OR = 0.99, 95% CI [0.58–1.71], p = .997) b. Smoking frequency:  i. T2: Further reduction from baseline (B = −0.42, SE = 0.07, p < .001). ii. T3: Still significantly reduced compared to baseline (B = −0.30, SE = 0.06, p < .001), but slightly higher than at T2. c. Smoking quantity: i. T2: Remained below baseline (B = −0.45, SE = 0.10, p < .001). ii. T3: Still significantly lower than baseline (B = −0.25, SE = 0.08, p = .003), though higher than at T1. No significant intervention effects at T2 or T3 for cessation, frequency, quantity. | 1. Breastfeeding/Lactation (42 days postpartum, Clinical observation form): Exclusive breastfeeding rate: Intervention: 62 (82.60%), Control: 46 (61.30%), p < 0.001. | 1. Sleep (42 days postpartum); Sleep patterns (SOL, WASO, TST, sleep efficiency): No significant between-group differences were observed. Sleep quality (PSQI): The dMBI-PI group showed a slightly greater reduction than controls (–1.12, 95% CI –2.25 to 0.01; p=.053), but this did not reach statistical significance. 2. Insomnia (42 days postpartum, ISI): Both groups showed sustained reductions in ISI scores from baseline. The mean decrease did not significantly differ between the intervention and control groups (between-group difference –1.12, 95% CI –2.57 to 0.33; p=0.13; Cohen’s d=0.12). Remission rates declined in both groups compared with earlier assessments, with no significant differences observed (OR 1.18, 95% CI 0.61–2.26; p=0.62). |
| OW; Overweight, OB; Obesity, NW; Normal weight, UW: Underweight, PA; Physical Activity, TAU; Treatment As Usual, BCC; Behaviour Change Communication, Wks; Weeks, SBP; Systolic Blood Pressure, DBP; Diastolic Blood Pressure, BP; Blood Pressure, HbA1c; Glycated haemoglobin, TC; Total cholesterol, HDL; High-density lipoprotein, TG; Triglycerides, MDD-W; Minimum Dietary Diversity for Women , CSUS; Cumulative Supplement Use Score, PPAQ; Pregnancy Physical Activity Questionnaire, IPAQ-SF; International Physical Activity Questionnaire – Short Form, PSQI; Pittsburgh Sleep Quality Index, ISI; Insomnia Severity Index, TST; Total Sleep Time, SOL; Sleep Onset Latency, WASO; Wake After Sleep Onset., SE; Sleep Efficiency, TWT; Total Wake Time, GWG; Gestational Weight Gain, GDM; Gestational Diabetes Mellitus, WC; Waist Circumference. | | | | | | | | | | | | | | | |

# Supplementary Table 7. Measures, Methods and Digitalization Category (Fully/Partially) of Included Lifestyle Interventions

| Measure | Method | Delivery/Format |  | |  |
| --- | --- | --- | --- | --- | --- |
| **PRECONCEPTION** | | | |  | |
| Hanafiah et al. (2022) | | | |  | |
| Nutrition | Adapted, locally validated Food Frequency Questionnaire (FFQ). | **Digitally** / Self-Reported | **Partially Digitalized** | |  |
| Physical Activity | International Physical Activity Questionnaire (IPAQ) | **Digitally** / Self-Reported |  |  |  |
| Anthropometrics | - Standardized WHO STEPS surveillance measurement (Weight, BMI, Hip circumference). - Anthropometric measurement and calculation using Asian-specific cut-offs (Waist circumference). | **In-Person:** Measured by study nurses. |  |  |  |
| Metabolic/Clinical Outcomes | - 10ml clinical blood sample (9-hour minimum fast, Blood sugar). - 10ml clinical blood sample (Cholesterol, LDL, HDL, Triglycerides). - Systolic and diastolic measurement following WHO STEPS. | **In-Person:** Clinical lab test performed by study nurses at health clinics. |  |  |  |
| Van Dijk et al. (2020) | | | |  | |
| Nutrition | Dietary Risk Score (DRS): Sum of scores from vegetable, fruit, and folic acid intake (Range: 0–9 for women (9 most unhealthy) | **Digital:** Calculated automatically based on online questionnaire responses. | **Fully Digitalized** | |  |
| Oostingh et al. (2020) | | | |  | |
| Nutrition | Dietary Risk Score (DRS): Grams of vegetables and pieces of fruit converted to a 0–3 risk scale. | **Digital:** Self-reported via online questionnaires. | **Fully Digitalized** | |  |
| Smoking & Alcohol | Lifestyle Risk Score (LRS): Weighting scale (0–9) based on daily cigarettes and weekly alcoholic beverages. | **Digital:** Self-reported via online questionnaires. |  |  |  |
| Fertility (Pregnancy Rates) | Cumulative Pregnancy Rate: Assessment of whether pregnancy occurred within 52 weeks. | **Digital/Phone:** Self-reported follow-up questionnaire. |  |  |  |
| **PREGNANCY** | | | |  | |
| Abd Rahman et al. (2022) | | | |  | |
| Nutrition | Food Frequency Questionnaire (FFQ) | **Digital:** Self-reported questionnaire. | **Partially Digitalized** | |  |
| Anemia (Hemoglobin Level) | Full Blood Count (FBC) via clinical lab analysis. | **In-Person:** Clinical blood draws conducted at the health clinics. |  |  |  |
| Chen et al. (2023) | | | |  | |
| Nutrition | - Self-monitoring of daily diet against IOM/ACOG standards. - 25-item 5-point Likert scale (Diet and Exercise behavior questionnaire). | **Digital:** Self-recorded through the MHW app interface.  **Digital / Self-Reported:** Electronic survey via the app. | **Partially Digitalized** | |  |
| Physical Activity | - Pregnancy Physical Activity Questionnaire (PPAQ). - Mi Band 5 (WAT): Wrist-worn fitness tracker measuring daily steps. - 25-item 5-point Likert scale (Diet and Exercise behavior questionnaire). | **In-Person / Digital:** Validated Mandarin survey completed by participants.  **Digital:** Passive data collection via Bluetooth.  **Digital** / Self-Reported**:** Electronic survey via the app. |  |  |  |
| Anthropometrics (Gestational Weight Gain) | Total GWG (pre-delivery minus pre-pregnancy) and weekly trajectory based on IOM guidelines. | **Mixed:** Pre-pregnancy EHR data + Self-recorded digital entries in the app. |  |  |  |
| Koeryaman et al. (2023) | | | |  | |
| Nutrition | 3-day food records & MDD-W survey | **Digital** (IG): Via the SISFORNUTRIMIL application.  CG: Via **paper-based** records. | **Partially Digitalized** | |  |
| Anthropometrics | Standard weight scales | **In-person**, at clinical assessment. |  |  |  |
| [Téoule](https://pubmed.ncbi.nlm.nih.gov/?term=%22T%C3%A9oule%20J%22%5bAuthor%5d) et al. (2024) | | | | | |
| Physical Activity | Huawei Band 6 Tracker: Measures average steps per day. | **Digital:** Data logged via app. | **Fully Digitalized** | |  |
| Anthropometrics (GWG) | Difference between pre-pregnancy weight and weight immediately before delivery. | **Clinical:** Data extracted from German maternity logs and medical records. |  |  |  |
| Sandborg et al. (2021) | | | |  | |
| Nutrition | Web-based 24-hour recall (Riksmaten FLEX) | **Digital:** Participants registered their intake over 3 days | **Partially Digitalized** | |  |
| Physical Activity | ActiGraph accelerometer & diary | **Digital:** Participants wore a wrist-mounted accelerometer for 7 consecutive days. |  |  |  |
| Anthropometrics | Bod Pod (Air-displacement plethysmography) | **In-person:** Measured at Linköping University Hospital. |  |  |  |
| Vadsaria et al. (2025) | | | | | |
| Nutrition | - Dietary Intake: Dietary Risk Score (DRS) - Micronutrient Intake: Cumulative Supplement Use Score (CSUS; 0-12), with higher scores indicating greater inadequacy. | **Digital/Paperless:** Assessment of food quantity and quality (0–3 scale).  **Digital/Paperless:** Questionnaires. | **Partially Digitalized** | |  |
| Anemia (Hemoglobin Level) | Full Blood Count (FBC) via clinical lab analysis. | **In-person:** Clinical lab tests. |  |  |  |
| Metabolic Outcomes |  | **In-person:** Clinical lab tests. |  |  |  |
| Valença et al. (2024) | | | | | |
| Nutrition | - 24-hour dietary recall (R24) (Dietary Intake) - DietPro software (using TA-CO, IBGE, & USDA tables) (Nutritional Analysis) | **Digital/Phone:** Conducted by researchers approximately every 10 days via phone call. | **Partially Digitalized** | |  |
| Metabolic Outcomes | Home-recorded capillary blood glucose | **In-person/Manual:** Patients recorded readings 4x daily; clinicians reviewed them at in-person visits. |  |  |  |
| Van Dijk et al. (2020) | | | | | |
| Nutrition | Dietary Risk Score (DRS): Sum of scores from vegetable, fruit, and folic acid intake (Range: 0–9 for women (9 most unhealthy) | **Digital**: Calculated automatically based on online questionnaire responses. | **Fully Digitalized** | |  |
| Zhou et al. (2025) | | | | | |
| Nutrition | Diet Management: 4 items, with a score range of 4-20 (series of dietary management practices implemented by pregnant women to achieve appropriate GWG) | **Digital** | **Partially Digitalized** | |  |
| Anthropometrics (GWG) | Gestational Weight Management Behavior Scale (4 dimensions) | **Digital:** Self-reported via the app. |  |  |  |
| Talebi et al. (2022) | | | | | |
| Physical Activity | Pregnancy Physical Activity Questionnaire (PPAQ): 32 items assessing 4 domains (home, community, work, sports). | **In-Person / Digital:** Self-reported questionnaire completed at baseline and after the 8-week intervention. | **Partially Digitalized** | |  |
| Anthropometrics (GWG, BMI) | - GWG (Seca scale): Change in body weight measured in kg. - BMI (Stadiometer): Calculated as weight (kg) divided by height (m) squared. | **In-Person:** Height measured via Stadiometer; weight via Seca scale. |  |  |  |
| Chen et al. (2024) | | | | | |
| Physical Activity | Mi Smart Band 5: Tracking daily steps with a specific goal of 8,500 steps/day. | **Digital:** Passive data collection via a wearable device. | **Fully Digitalized** | |  |
| Anthropometrics (GWG) | Calculated as body weight at hospital admission for delivery minus pre-pregnancy weight. | Extracted from hospital records and the 10 scheduled prenatal checkups. |  |  |  |
| Kalmbach et al. (2020) | | | | | |
| Sleep | - Pittsburgh Sleep Quality Index (PSQI): Measures duration, latency, and disturbances. - PSQI Item: Self-reported habitual sleep duration in hours. - Presleep Arousal Scale Cognitive (PSAS-C): Measures trait tendency for racing thoughts at night. | **Digital:** Self-reported via online surveys. | **Fully Digitalized** | |  |
| Insomnia | Insomnia Severity Index (ISI): 7-item scale (Score 0-28). Clinically significant >=10. | **Digital:** Self-reported via online surveys (Qualtrics) |  |  |  |
| Sun et al. (2021) | | | | | |
| Sleep | Pittsburgh Sleep Quality Index (PSQI) | **Digital:** Self-reported via smartphone-assisted survey. | **Fully Digitalized** | |  |
| Van Dijk et al. (2024) | | | | | |
| Smoking | Smoking Behavior: Self-reported status (yes/no), frequency (days/week), and quantity (cigarettes/week). | **Digital:** Online questionnaires. | **Fully Digitalized** | |  |
| Wang et al. (2025) | | | | | |
| Sleep | Consensus Sleep Diary-Core: 1 week of data (SOL, WASO, TST, Efficiency). | **Digital:** Self-recorded daily entries via web survey. | **Fully Digitalized** | |  |
| Insomnia | Insomnia Severity Index (ISI) | **Digital:** Web-based self-assessed surveys. |  | |  |
| Yang et al. (2023) | | | | | |
| Anthropometrics (GWG) | Tracking excessive weight gain during pregnancy. | **Clinical Observation:** Data extracted from hospital electronic records. | **Partially Digitalized** | |  |
| Self-Efficacy | General Self-efficacy Scale (GSES): 10 items (Score 10–40). | **In-person / Paper** |  |  |  |
| Felder et al. (2020) | | | | | |
| Sleep | - Quality: Pittsburgh Sleep Quality Index (PSQI). - Efficiency/Duration: 7-Day Sleep Diaries: Calculated as (Time asleep / Time in bed)*100. |  | **Fully Digitalized** | |  |
| Insomnia | Insomnia Severity Index (ISI) | **Digital:** Self-reported via Qualtrics/REDCap surveys. |  |  |  |
| Wi et al. (2025) | | | | | |
| Sleep | - Quality: Pittsburgh Sleep Quality Index (PSQI). - Wrist Actigraphy: 7 nights of continuous monitoring (TST, SE, SOL, WASO). | **Digital:** REDCap surveys.  **Wearable:** Actiwatch Spectrum Plus device. | **Fully Digitalized** | |  |
| Gonzalez-Plaza et al. (2022) | | | | | |
| Physical Activity | - IPAQ-SF: Calculated as MET-minutes per week (categories: low, moderate, high). - Step Count: Mi Band 2 | **Digital** / Self-Report | **Partially Digitalized** | |  |
| Anthropometrics (GWG) | GWG: Measured as the difference between pre-pregnancy weight and weight at 35–37 weeks. | **In-Person:** Measured by a midwife using a Seca 704 scale; categorized by IOM guidelines. |  |  |  |
| Gestational Diabetes Mellitus (GDM) |  | **In-Person / EMR:** Data obtained by midwives and clinical history review. |  |  |  |
| Bernando et al. (2024) | | | | | |
| Physical Activity | PPAQ (Pregnancy Physical Activity Questionnaire) | **Digital/Paper:** Self-reported questionnaire. | **Partially Digitalized** | |  |
| Anthropometrics (GWG) | GWG: Difference between weight at 8 weeks and baseline (IOM goal: <=0.22kg/week) + Motion trackers | **In-Person:** Measured by a blinded nurse using a Tanita BC-545 digital scale.  **Wearable:** Automatically downloaded via the Phoenix® platform. |  |  |  |
| **POSTPARTUM** | | | | | |
| Chen et al. (2024) | | | | | |
| Physical Activity | Mi Smart Band 5: Tracking daily steps with a specific goal of 8,500 steps/day. | **Digital:** Passive data collection via a wearable device. | **Fully Digitalized** | |  |
| Anthropometrics (Postpartum Weight Retention) | Calculated as 6-month postpartum weight minus pre-pregnancy weight. | **Digital:** Self-reported via questionnaire at the 6-month follow-up. |  |  |  |
| Sun et al. (2021) |  |  |  | |  |
| Sleep | Pittsburgh Sleep Quality Index (PSQI) | **Digital:** Self-reported via smartphone-assisted survey. | **Fully Digitalized** | |  |
| Van Dijk et al. (2024) | | | | | |
| Smoking | Smoking Behavior: Self-reported status (yes/no), frequency (days/week), and quantity (cigarettes/week). | **Digital:** Online questionnaires. | **Fully Digitalized** | |  |
| Wang et al. (2025) | | | | | |
| Sleep | Consensus Sleep Diary-Core: 1 week of data (SOL, WASO, TST, Efficiency). | **Digital:** Self-recorded daily entries via web survey. | **Fully Digitalized** | |  |
| Insomnia | Insomnia Severity Index (ISI) | **Digital:** Web-based self-assessed surveys. |  |  |  |
| Yang et al. (2023) | | | | | |
| Anthropometrics (GWG) | Tracking excessive weight gain during pregnancy. | **Clinical Observation:** Data extracted from hospital electronic records. | **Partially Digitalized** | |  |
| Self-Efficacy | General Self-efficacy Scale (GSES): 10 items (Score 10–40). | **In-person / Paper** |  |  |  |
| Breastfeeding | Breastfeeding rate | **Clinical Observation** |  |  |  |
| Bijlholt et al. (2021) | | | | | |
| Nutrition | - Dietary Intake: Food Frequency Questionnaire (FFQ) - TFEQ-R18: Measures restrained, uncontrolled, and emotional eating. | **Digital:** Online questionnaire. | **Partially Digitalized (face-to-face coaching)** | |  |
| Physical Activity | IPAQ + Wearable (Withings GO activity tracker and Withings Body+ smart scale) | **Digital:** Online questionnaire. |  |  |  |
| Lim et al. (2021) | | | | | |
| Nutrition | Dietary Intake: 3-day food diaries. | **Hybrid:** Mailed to participants, then manually submitted at the clinic. | **Partially Digitalized** | |  |
| Physical Activity | Step counting and manual activity logs. | **Digital:** Synced or manually entered via the nBuddy app. |  |  |  |
| Anthropometrics | - Postpartum Weight Retention/Loss: Comparison of 4-month postpartum weight to first-trimester weight. - Waist Circumference | **In-Person:** Measured using a calibrated Seca 799 digital scale.  **In-Person:** Clinical measurements taken by research staff. |  |  |  |
| Metabolic Outcomes | Blood Pressure | **In-Person:** Clinical measurements taken by research staff. |  |  |  |
| Potzel et al. (2022) | | | | | |
| Nutrition | 4-day food/drink diary (mean calorie, fiber, and fat intake) analyzed via PRODI® 6 Basis software. | **Digital** | **Partially Digitalized** | |  |
| Physical Activity | - Cardiopulmonary exercise testing (CPET) to measure peak oxygen uptake (VO2peak). - Step Counts | **In-Person:** Stepwise ergospirometry on a bicycle ergometer.  **Digital/Wearable:** Garmin vívosmart HR®. |  |  |  |
| He et al. (2025) | | | | | |
| Physical Activity | Muscle Strength: Pelvic Floor Surface EMG, evaluating deep/superficial layers (fast/slow muscle fibers) via Glazer method. | **In-Person:** Biofeedback device (Thinking Technology SA9800). | **Partially Digitalized** | |  |
| Urinary Incontinence | Incontinence Questionnaire Short Form (ICIQ-SF) (Frequency/amount of leakage and impact on quality of life (Score 0–21). | **Digital/Paper:** Self-reported. |  |  |  |
| Badon et al. (2025) | | | | | |
| Physical Activity | - PPAQ: Sports and exercise domain, including a "MomZing-specific" subscale. - Device-Measured PA/dm-MVPA (Moderate-to-vigorous physical activity duration.) | **Wearable:** ActiGraph GT3X+ accelerometer.  **Digital:** Online surveys. | **Fully Digitalized** | |  |
| Sleep | PSQI | **Digital:** Online surveys. |  |  |  |
| Taylor et al. (2022) | | | | | |
| Nutrition | Diet Quality: ARFS (Australian Recommended Food Score). | **Digital:** Self-administered online survey. | **Fully Digitalized** | |  |
| Physical Activity | Godin Leisure-Time Exercise Questionnaire: Measures light, moderate, and vigorous activity. | **Digital:** Online survey; |  |  |  |
| Anthropometrics | Postpartum Weight Retention/Loss & BMI: Self-reported weight (kg) and height (m). | **Digital:** Collected via Qualtrics online surveys. |  |  |  |
| Nicklas et al., (2024) | | | | | |
| Nutrition | Dietary Intake: 2005 Block FFQ, estimating habitual dietary intake. | **Digital:** Administered via NutritionQuest (REDCap). | **Partially Digitalized** | |  |
| Physical Activity | - PPAQ (Adapted) - Step Counts | **Digital:** Online surveys via REDCap.  **Wearable:** Data passively transmitted from a Fitbit via Bluetooth to the FAB app. |  |  |  |
| Anthropometrics | Postpartum Weight Retention/Loss: Measured at 12 months vs. self-reported pre-pregnancy and first postpartum weight. | **In-Person:** Measured twice by trained staff using a SECA 360 scale. |  |  |  |
| Metabolic Outcomes | Fasting blood samples for glucose, insulin, HbA1c, lipids, adiponectin, and hsCRP. | **In-Person:** Clinical blood draws. |  |  |  |
| Van Uytsel et al. (2022) | | | | | |
| Nutrition | - Dietary Intake: Food Frequency Questionnaire (FFQ). - TFEQ-R18: Assesses restrained, uncontrolled, and emotional eating. | **Digital:** Self-reported online questionnaire via Castor eCRF. | **Partially Digitalized** | |  |
| Physical Activity | IPAQ | **Digital:** Self-reported online questionnaire. |  |  |  |
| Anthropometrics | - Tanita MC 780 SMA (BIA): Measures weight and fat % using 3 frequencies (5, 50, 250 kHz). - Seca 201 Tape: Waist and hip circumference. | **In-Person:** Repeated 3 times per visit. |  |  |  |
| Herring et al. (2024) | | | | | |
| Anthropometrics | Weight | **In-Person/Digital:** Initially measured via calibrated SECA scales; transitioned to BodyTrace digital scales during COVID-19. | **Partially Digitalized** | |  |
| Metabolic/Clinical Outcomes | - Fasting blood samples for Lipids (HDL, LDL, Triglycerides) and HbA1c. - Blood Pressure: GE Dinamap ProCare 400. | **In-Person:** Samples collected.  **In-Person:** Conducted after a 5-minute rest; supplemented by EHR data during the pandemic. |  |  |  |
| Kalmbach et al. (2020) | | | | | |
| Sleep | - Pittsburgh Sleep Quality Index (PSQI): Measures duration, latency, and disturbances. - PSQI Item: Self-reported habitual sleep duration in hours. - Presleep Arousal Scale Cognitive (PSAS-C): Measures trait tendency for racing thoughts at night. | **Digital:** Self-reported via online surveys. | **Fully Digitalized** | |  |
| Insomnia | Insomnia Severity Index (ISI): 7-item scale (Score 0-28). Clinically significant >=10. | **Digital:** Self-reported via online surveys (Qualtrics) |  |  |  |

# Supplementary Table 8. PRISMA 2020 checklist

| **Section and Topic** | **Item #** | **Checklist item** | **Location where item is reported** |
| --- | --- | --- | --- |
| **TITLE** | | |  |
| Title | 1 | Identify the report as a systematic review. | “Effectiveness of digital tools on lifestyle & health-related outcomes across the full continuum of the maternal journey: A systematic review” **Page 2.** |
| **ABSTRACT** | | |  |
| Abstract | 2 | See the PRISMA 2020 for Abstracts checklist. | **Page 2.** |
| **INTRODUCTION** | | |  |
| Rationale | 3 | Describe the rationale for the review in the context of existing knowledge. | “Although several primary studies…preconception to postpartum” **Page 4.** |
| Objectives | 4 | Provide an explicit statement of the objective(s) or question(s) the review addresses. | “Hence, this systematic review aims to…to postpartum” **Page 3.** |
| **METHODS** | | |  |
| Eligibility criteria | 5 | Specify the inclusion and exclusion criteria for the review and how studies were grouped for the syntheses. | “Studies’ eligibility followed…Non-randomized studies were excluded” **Page 4.** |
| Information sources | 6 | Specify all databases, registers, websites, organisations, reference lists and other sources searched or consulted to identify studies. Specify the date when each source was last searched or consulted. | “Two independent researchers …assessed via Cochrane Library.”  “Searches were restricted to studies published between 1st of January 2020 and 28th of May 2025” **Page 5.** |
| Search strategy | 7 | Present the full search strategies for all databases, registers and websites, including any filters and limits used. | **Supplementary Material 1** |
| Selection process | 8 | Specify the methods used to decide whether a study met the inclusion criteria of the review, including how many reviewers screened each record and each report retrieved, whether they worked independently, and if applicable, details of automation tools used in the process. | Supplementary Table 1. PICOS criteria |
| Data collection process | 9 | Specify the methods used to collect data from reports, including how many reviewers collected data from each report, whether they worked independently, any processes for obtaining or confirming data from study investigators, and if applicable, details of automation tools used in the process. | “For each study…can be found in detail in the Supplementary Material 1” **Pages 5-6.** |
| Data items | 10a | List and define all outcomes for which data were sought. Specify whether all results that were compatible with each outcome domain in each study were sought (e.g. for all measures, time points, analyses), and if not, the methods used to decide which results to collect. | “When an outcome…can be found in detail in the Supplementary Material 1” **Page 6.** |
|  | 10b | List and define all other variables for which data were sought (e.g. participant and intervention characteristics, funding sources). Describe any assumptions made about any missing or unclear information. | “Outcomes: Clinical or behavioral outcomes…unless linked to lifestyle factors” **Page 5.** |
| Study risk of bias assessment | 11 | Specify the methods used to assess risk of bias in the included studies, including details of the tool(s) used, how many reviewers assessed each study and whether they worked independently, and if applicable, details of automation tools used in the process. | “The quality and risk…is presented in Supplementary Material 1” **Page 6.** |
| Effect measures | 12 | Specify for each outcome the effect measure(s) (e.g. risk ratio, mean difference) used in the synthesis or presentation of results. | As no meta-analysis was performed, formal quantitative effect measures (e.g., risk ratios, odds ratios, or mean differences) were not calculated. Results were presented descriptively based on the reported outcomes of the included studies. |
| Synthesis methods | 13a | Describe the processes used to decide which studies were eligible for each synthesis (e.g. tabulating the study intervention characteristics and comparing against the planned groups for each synthesis (item #5)). | Due to heterogeneity among data, alongside the poor methodological quality of the studies included, a meta-analysis was not appropriate. Therefore, a qualitative synthesis approach was used. Studies were grouped according to the maternity stage (preconception, pregnancy, postpartum) but also lifestyle factors (e.g. nutrition, physical activity) and findings were summarised and compared descriptively across studies. |
|  | 13b | Describe any methods required to prepare the data for presentation or synthesis, such as handling of missing summary statistics, or data conversions. | N/A |
|  | 13c | Describe any methods used to tabulate or visually display results of individual studies and syntheses. | N/A |
|  | 13d | Describe any methods used to synthesize results and provide a rationale for the choice(s). If meta-analysis was performed, describe the model(s), method(s) to identify the presence and extent of statistical heterogeneity, and software package(s) used. | N/A |
|  | 13e | Describe any methods used to explore possible causes of heterogeneity among study results (e.g. subgroup analysis, meta-regression). | N/A |
|  | 13f | Describe any sensitivity analyses conducted to assess robustness of the synthesized results. | N/A |
| Reporting bias assessment | 14 | Describe any methods used to assess risk of bias due to missing results in a synthesis (arising from reporting biases). | N/A |
| Certainty assessment | 15 | Describe any methods used to assess certainty (or confidence) in the body of evidence for an outcome. | N/A |
| **RESULTS** | | |  |
| Study selection | 16a | Describe the results of the search and selection process, from the number of records identified in the search to the number of studies included in the review, ideally using a flow diagram. | “Figure 1. PRISMA (Preferred Reporting Items for Systematic Reviews and Meta-Analyses) Flowchart” **Page 8.** |
|  | 16b | Cite studies that might appear to meet the inclusion criteria, but which were excluded, and explain why they were excluded. | Supplementary Material 1, Supplementary Table 3. Reasons for Exclusion. **Pages 5-6.** |
| Study characteristics | 17 | Cite each included study and present its characteristics. | Supplementary Material 1, (a) Supplementary Table 4. Preconception, (b) Supplementary Table 5. Pregnancy, (c) Supplementary Table 6. Postpartum. **Pages 8-31.** |
| Risk of bias in studies | 18 | Present assessments of risk of bias for each included study. | Supplementary Material 1, Supplementary Figure 2. Traffic-Light Plot of Included Studies (n = 31). **Page 7.** |
| Results of individual studies | 19 | For all outcomes, present, for each study: (a) summary statistics for each group (where appropriate) and (b) an effect estimate and its precision (e.g. confidence/credible interval), ideally using structured tables or plots. | Supplementary Material 1, (a) Supplementary Table 4. Preconception, (b) Supplementary Table 5. Pregnancy, (c) Supplementary Table 6. Postpartum. **Pages 8-31.** |
| Results of syntheses | 20a | For each synthesis, briefly summarise the characteristics and risk of bias among contributing studies. |  |
|  | 20b | Present results of all statistical syntheses conducted. If meta-analysis was done, present for each the summary estimate and its precision (e.g. confidence/credible interval) and measures of statistical heterogeneity. If comparing groups, describe the direction of the effect. | “Results” **Pages 8-15.** |
|  | 20c | Present results of all investigations of possible causes of heterogeneity among study results. | N/A |
|  | 20d | Present results of all sensitivity analyses conducted to assess the robustness of the synthesized results. | N/A |
| Reporting biases | 21 | Present assessments of risk of bias due to missing results (arising from reporting biases) for each synthesis assessed. | N/A |
| Certainty of evidence | 22 | Present assessments of certainty (or confidence) in the body of evidence for each outcome assessed. | N/A |
| **DISCUSSION** | | |  |
| Discussion | 23a | Provide a general interpretation of the results in the context of other evidence. | “Our findings are in line…accessibility for all populations” **Pages 16-17.** |
|  | 23b | Discuss any limitations of the evidence included in the review. | “Limitations” **Page 18.** |
|  | 23c | Discuss any limitations of the review processes used. | “Limitations” **Page 18.** |
|  | 23d | Discuss implications of the results for practice, policy, and future research. | “Future Directions” **Page 18.** |
| **OTHER INFORMATION** | | |  |
| Registration and protocol | 24a | Provide registration information for the review, including register name and registration number, or state that the review was not registered. | “Study Design” **Page 4.** |
|  | 24b | Indicate where the review protocol can be accessed, or state that a protocol was not prepared. | <https://www.crd.york.ac.uk/PROSPERO/view/CRD420251075108> PROSPERO registry database |
|  | 24c | Describe and explain any amendments to information provided at registration or in the protocol. | N/A |
| Support | 25 | Describe sources of financial or non-financial support for the review, and the role of the funders or sponsors in the review. | N/A |
| Competing interests | 26 | Declare any competing interests of review authors. | N/A |
| Availability of data, code and other materials | 27 | Report which of the following are publicly available and where they can be found: template data collection forms; data extracted from included studies; data used for all analyses; analytic code; any other materials used in the review. | Supplementary Material 1.  Any other materials used in the review: (a) PICOS criteria, (b) Search strategy, (c) Reasons for Exclusion, (d) Traffic-Light Plot of Included Studies (n = 31), (e) Preconception/Pregnancy/Postpartum characteristics/outcomes of the studies |
